# CAF-derived miR-642a-3p supports migration, invasion, and EMT of hepatocellular carcinoma cells by targeting SERPINE1

**DOI:** 10.7717/peerj.18428

**Published:** 2024-11-11

**Authors:** Shuo Zhang, Gang Cao, Shuijie Shen, Yu Wu, Xiying Tan, Xiaoyan Jiang

**Affiliations:** 1 Department of Pharmacy, Nantong Hospital of Traditional Chinese Medicine, Nantong, China; 2 Office of the Dean, Nantong Maternal and Child Health Care Hospital, Nantong, China; 3 Department of Science and Education, Nantong Hospital of Traditional Chinese Medicine, Nantong, China; 4 Department of Pharmacy, Affiliated Hospital of Nanjing University of Chinese Medicine, Nanjing, China

**Keywords:** Hepatocellular carcinoma, Cancer-associated fibroblasts, miR-642a-3p, SERPINE1, Invasion, EMT

## Abstract

**Background:**

Cancer-associated fibroblasts (CAFs) and hepatocellular carcinoma (HCC) cells interact to promote HCC progression, but the underlying mechanisms remain unclear. Serpin family E member 1 (SERPINE1) has conflicting roles in HCC, and microRNAs (miRNAs) are known to regulate tumor progression through intercellular communication. Therefore, we investigated the potential involvement of miRNA/SERPINE1 axis in crosstalk between CAFs and HCC cells.

**Methods:**

In this study, candidate miRNAs targeting *SERPINE1* 3′ UTR were predicted using multiple miRNA databases. The miRNAs and *SERPINE1* mRNA expression in Huh7 cells was assessed after co-culture with CAFs using RT-qPCR. Huh7 cell proliferation and invasion were detected after *SERPINE1* siRNA. The functions of the CAF-derived miR-642a-3p/SERPINE1 axis in HCC cells were examined using CCK-8, wound healing, transwell assays, western blot, and dual-luciferase reporter assays. Moreover, a orthotopic xenograft model was used to investigate the contribution of miR-642a-3p knockdown in HCC.

**Results:**

*SERPINE1* mRNA expression decreased, while miR-642a-3p expression increased in Huh7 cells co-cultured with CAFs. *SERPINE1* knockdown enhanced Huh7 cell proliferation and invasion as well as miR-642a-3p expression. miR-642a-3p overexpression promoted migration, invasion, and epithelial-mesenchymal transition (EMT) in Huh7 cells by targeting SERPINE1, while miR-642a-3p knockdown yielded the opposite effect. Rescue experiments confirmed that *SERPINE1* knockdown attenuated the inhibitory effects of miR-642a-3p knockdown on migration, invasion, and EMT in Huh7 cells. Importantly, miR-642a-3p knockdown suppressed growth and EMT in orthotopic liver tumors.

**Conclusion:**

CAF-derived miR-642a-3p/SERPINE1 axis facilitated migration, invasion, and EMT in the HCC cells, suggesting miR-642a-3p/SERPINE1 axis can be a potential therapeutic target for HCC.

## Introduction

Liver cancer is a prevalent malignant neoplasm ranking as the fourth leading cause of cancer-related mortality (Ghafouri-Fard et al., 2021). Globally, in 2020, the incidence of new liver cancer cases and associated deaths was 9.5 and 8.7 per 100,000 individuals, respectively, with both rates exhibiting annual increases (Rumgay et al., 2022). Hepatocellular carcinoma (HCC) is the most common primary liver malignancy and constitutes approximately 80–90% of primary liver cancer (Ladd et al., 2024; Phoolchund & Khakoo, 2024; Sankar et al., 2024). The primary cause of HCC is chronic liver disease, including cirrhosis and chronic hepatitis (Sankar et al., 2024). Alcohol usage, non-alcoholic steatohepatitis (NASH), hepatitis B virus (HBV), and hepatitis C virus (HCV) can all result in chronic hepatitis (Phoolchund & Khakoo, 2024; Sankar et al., 2024). Despite significant advancements in HCC treatment over recent decades, early detection remains challenging. Consequently, more than 70% of patients are diagnosed at advanced disease stages, resulting in a markedly low five-year survival rate following surgical intervention (Hou et al., 2022; Vogel et al., 2022; Zhou & Song, 2021). Furthermore, chemotherapy resistance and recurrent disease are major contributors to the poor prognosis of HCC patients (Ganesan & Kulik, 2023; Hou et al., 2022). Consequently, the identification of biomarkers associated with HCC initiation and progression is crucial for enhancing prevention, diagnostic accuracy, therapeutic efficacy, and prognostication (Lv & Sun, 2024).

During tumorigenesis, a heterogeneous population of cells congregates around cancer tissues, forming a distinctive microenvironment known as the tumor microenvironment (TME) (Basak et al., 2023; Hoekstra et al., 2024; Yu, Huang & Guo, 2024). These cells are recruited to fulfill pro-tumorigenic functions, enabling cancer cells to evade immune surveillance and establish a tumor niche (Hoekstra et al., 2024; Yasuda & Wang, 2024; Yu, Huang & Guo, 2024). Cancer-associated fibroblasts (CAFs), a predominant component of the TME, exert a multitude of oncogenic effects within tumor tissues, including alterations in tumor metabolism and immune reprogramming, facilitation of immune evasion, enhancement of drug resistance, and modulation of the TME (Arpinati, Carradori & Scherz-Shouval, 2024; Jing et al., 2024; Ye et al., 2024). Through the secretion of growth factors, immunomodulatory molecules, and extracellular matrix proteins, CAFs remodel the extracellular matrix and TME, thereby promoting metastasis, immune escape, and therapeutic resistance in tumors (Arpinati, Carradori & Scherz-Shouval, 2024; Biffi & Tuveson, 2021; Helms, Onate & Sherman, 2020; Miyai et al., 2020). Consequently, CAFs have emerged as a focal point for clinical and preclinical investigations (Caligiuri & Tuveson, 2023). In HCC, activated CAFs interact with HCC cells, expressing various pro-proliferative and pro-invasive factors, thereby creating a permissive microenvironment for HCC cell proliferation, growth, invasion, and migration (Schneider et al., 2024; Shang et al., 2024). Moreover, characterizing distinct CAF clusters has demonstrated prognostic value in HCC, offering a novel therapeutic approach (Yu et al., 2022).

In HCC, CAFs stimulate serpin family E member 1 (SERPINE1) expression in tumor-associated macrophages (TAMs), thereby promoting the malignant progression of HCC cells through epithelial-mesenchymal transition (EMT) (Chen et al., 2021). As a member of the serine protease inhibitor family, SERPINE1 is a critical regulator of extracellular matrix remodelling (Higgins et al., 2019; Kong et al., 2021). This protein participates in diverse physiological processes, including metabolism, inflammation, angiogenesis, cancer, and aging (Sillen & Declerck, 2021; Wang et al., 2023a). SERPINE1 interacts with biological ligands, such as vitronectin and cell surface receptors, to engage in pericellular proteolysis, tissue remodeling, and cell migration (Sillen & Declerck, 2021). SERPINE1 has been implicated in multiple facets of cancer progression, including proliferation, migration, invasion, EMT, angiogenesis, and drug resistance (Nagy, Munkácsy & Győrffy, 2021; Su et al., 2024a; Teng et al., 2021). Overexpressed in various cancers, including gastric (Chen et al., 2022; Tutunchi et al., 2021), lung (Hong et al., 2022; Setiawan et al., 2024), and colon (Wang et al., 2023b) cancers, SERPINE1 is classified as a pan-oncogene and is associated with poor prognosis (Ju et al., 2024). However, the functional role of SERPINE1 in HCC remains controversial (Jin et al., 2020). While some studies report high SERPINE1 expression in HCC, promoting tumor progression (Zhang et al., 2021), others suggest a tumor-suppressive role for SERPINE1, with overexpression inhibiting HCC cell invasion (Wang et al., 2016). This complexity underscores the need for further investigation into the multifaceted functions of SERPINE1 in HCC.

Non-coding RNAs (ncRNAs) play a pivotal role in controlling cell communication within TME (Szymanowska et al., 2023). They regulate tumor cell proliferation, apoptosis, metastasis, and drug resistance, and are therefore considered as potential cancer markers (Chakrabortty et al., 2023; Iaccarino & Klapper, 2021). As a subset of ncRNAs, microRNAs (miRNAs) are single strand molecules with 20–24 nucleotides that bind to mRNA to control the expression of post-transcriptional genes (Chakrabortty et al., 2023). Dysregulation of miRNA expression is associated with cancer progression (Chakrabortty et al., 2023; Parsa-Kondelaji et al., 2023). While aberrant expression of many miRNAs, including miR-22 (Hu et al., 2023) and miR-17-5p (Zhou et al., 2024), has been linked to HCC progression, the involvement of miRNAs in CAFs-HCC communication remains relatively unexplored (Su et al., 2024b).

The purpose of this study was to investigate the role of CAF-derived miRNA on HCC cells through regulating SERPINE1. First, we screen candidate miRNAs targeting SERPINE1 through multiple miRNA databases (StarBase, miRwalk, TargetScan, and miRDB). Subsequently, we analyzed whether CAFS-derived miR-642a-3p targeted SERPINE1 *via* co-culture of CAFs and HCC cells, real-time quantitative PCR (RT-qPCR), and dual-luciferase reporter assays. Finally, the functional significance of miR-642a-3p/SERPINE1 axis in HCC cells was explored *in vitro* and *in vivo* to identify novel therapeutic targets for HCC.

## Materials and Methods

### Cell culture

Huh7 cells (a human hepatoma cell line) and human hepatocellular CAFs were obtained from Jiangsu KeyGEN Biotechnology Co., Ltd. (KeyGEN BioTECH, Nanjing, China) and Shanghai Fusheng Industrial Co., Ltd. (Shanghai, China), respectively. The cells were cultured in Dulbecco’s Modified Eagle’s Medium (DMEM) (KeyGEN BioTECH) supplemented with 10% fetal bovine serum (FBS) (Gibco, USA) and 1% penicillin-streptomycin (P/S) (KeyGEN BioTECH) in a humidified incubator at 37 °C with 5% CO_2_.

Huh7 cells and CAFs were co-cultured in a transwell system. CAFs were seeded in the upper chamber of the transwell, while Huh7 cells were seeded in the lower chamber. A transwell without CAFs served as the control. Co-culture was maintained for 48 h.

### MiRNAs bioinformatics prediction

The miRNAs binding to *SERPINE1* 3′ untranslated region (UTR) were predicted using the starBase (https://rnasysu.com/encori/), miRwalk (http://mirwalk.umm.uni-heidelberg.de/), TargetScan (https://www.targetscan.org/vert_80/), and miRDB (https://mirdb.org/) databases. The predicted miRNAs were intersected by Venn diagram.

### Cell transfection

Huh7 cells were transfected with miR-642a-3p mimics, miR-642a-3p inhibitor, or *SERPINE1* siRNA (si-SERPINE1) (General Biology, Anhui, China) for 48 h using Lipofectamine™ 3000 transfection reagent (Invitrogen, USA) according to the manufacturer’s protocol. Briefly, 125 µL Opti-MEM medium containing 100 pmol *SERPINE1* siRNA (20 µM) or miR-642a-3p mimics/inhibitor (20 µM) and 5 µL P3000™ reagent was gently mixed. Similarly, 125 µL Opti-MEM medium and 3.75 µL Lipofectamine™ 3000 reagent were combined. The diluted Lipofectamine™ 3000 reagent was then added to the diluted siRNA mixture, gently mixed, and incubated at room temperature for 10-15 min. Once the cell confluence reached 70–80% in the 6-well plate, 250 µL of the transfection mixture was added, and the cells were cultured at 37 °C for 48 h. Negative control groups included cells transfected with miR-642a-3p mimics negative control (mimics NC), miR-642a-3p negative inhibitor (inhibitor NC), or siRNA negative control (si-NC). Each experimental group was performed in triplicate. The sequences of the miR-381-3p mimics, inhibitor, *SERPINE1* siRNA, and respective negative controls are provided in Table 1.

**Table 1:** The sequences of the miR-642a-3p mimics, inhibitor, SERPINE1 siRNA, and respective NCs.

**Name**	Sequence (5′ → 3′)
miR-642a-3p mimics	F: AGACACAUUUGGAGAGGGAACC
	R: GGUUCCCUCUCCAAAUGUGUCU
mimics NC	F: UCACAACCUCCUAGAAAGAGUAGA
	R: UCUACUCUUUCUAGGAGGUUGUGA
miR-642a-3p inhibitor	GGUUCCCUCUCCAAAUGUGUCU
Inhibitor NC	UCUACUCUUUCUAGGAGGUUGUGA
SERPINE1 siRNA#1	F: GGAAAGGAGCCGUGGACCATT
	R: UGGUCCACGGCUCCUUUCCTT
SERPINE1 siRNA#2	F: CGACAUGUUCAGACAGUUUTT
	R: AAACUGUCUGAACAUGUCGTT
SERPINE1 siRNA#3	F: GGCCAUGGAACAAGGAUGATT
	R: UCAUCCUUGUUCCAUGGCCTT
siRNA NC	F: UUCUCCGAACGUGUCACGUTT
	R: ACGUGACACGUUCGGAGAATT

### CCK-8 assay

Huh7 cell proliferation was quantified using the CCK-8 Cell Proliferation Detection Kit (KeyGEN BioTECH). Briefly, Huh7 cells transfected with either si-NC or si-SERPINE1 were cultured in 96-well plates for 48 h. Subsequently, 10 µL of CCK-8 reagent was added to each well, followed by incubation at 37 °C for 2 h. Absorbance values were measured at 450 nm using an ELx800 Microplate Reader (BioTek, Winooski, VT, USA).

### Wound healing assay

Huh7 cells were seeded into six-well cell culture plates at a density of 1 × 10^5^ cells/mL and incubated overnight. A sterile pipette tip was employed to create a scratch wound in each well. Unattached cells were removed by washing with 1× PBS, followed by the replacement of the culture medium with fresh medium. The cells were then concurrently transfected. After a 48-hour incubation period, images were captured at 100× magnification using an IX51 microscope (Olympus, Tokyo, Japan). Wound width was measured at 0 h (a) and 48 h (b), and the wound healing ratio [(*a* − *b*)/*a* × 100%] was calculated to assess migratory capacity.

### Transwell assay

To assess Huh7 cell invasion, a 24-well transwell chamber (Corning Incorporated, USA) coated with Matrigel (BD, Franklin Lakes, NJ, USA) was employed. The cells were seeded at a density of 1 × 10^5^ cells/mL within the transwell chamber. The lower chamber was filled with 500 µL of DMEM supplemented with 10% FBS. Following a 48-hour incubation period, the cells on the upper surface of the membrane were removed using cotton swabs. The cells on the lower surface of the membrane were stained with 0.1% crystal violet (Sigma) for 30 min at 37 °C, washed twice with 1× PBS, imaged using an IX51 microscope (Olympus, Japan), and quantified.

### Total RNA extraction and RT-qPCR

Total RNA was isolated using TRIzol Reagent (Invitrogen, USA) according to the manufacturer’s protocol. RNA integrity was assessed *via* agarose gel electrophoresis, and concentration and purity were determined using a Nano100 spectrophotometer (Hangzhou Allsheng Instruments Co., Ltd., Hangzhou, China). First-strand cDNA synthesis was performed using the PrimeScript™ RT reagent Kit (Takara, Shiga, Japan) with total RNA as a template. For miRNA expression analysis, reverse transcription was conducted with a Bulge-Loop™ miRNA RT-PCR Starter Kit (RiboBio). *GAPDH* or *U6* served as the endogenous control gene. Quantitative PCR was performed using SYBR Green PCR Mix (Takara) on a StepOnePlus Real-Time PCR System (ABI, USA). Relative gene mRNA and miRNA expression levels were calculated using the 2^−ΔΔ*Ct*^ method based on three biological replicates with three technical replicates each. The primers sequences for miR-642a-3p, miR-3135a, miR-449b-5p, miR-642a-3p, miR-3135a, *SERPINE1*, *GAPDH*, and *U6* were synthesized by General Biosystems (Anhui) Co., Ltd. (Anhui, China) and are listed in Table 2.

**Table 2:** The primer sequences of the genes.

**Name**	Sequence (5′ → 3′)
miR-449b-5p	F:ACACTCCAGCTGGGAGGCAGTGTATTGTTA
	R:TGGTGTCGTGGAGTCG
miR-544b	F: ACACTCCAGCTGGGACCTGAGGTTGTGCAT
	R: TGGTGTCGTGGAGTCG
miR-642a-3p	F: ACACTCCAGCTGGGAGACACATTTGGAGAG
	R: TGGTGTCGTGGAGTCG
miR-2116-3p	F: ACACTCCAGCTGGGCCTCCCATGCCAAGA
	R: TGGTGTCGTGGAGTCG
miR-3135a	F: ACACTCCAGCTGGGTGCCTAGGCTGAGACT
	R: TGGTGTCGTGGAGTCG
SERPINE1	F: GGTGCTGGTGAATGCCCTCTAC
	R: TGCTGCCGTCTGATTTGTGGAA
GAPDH	F: AGATCATCAGCAATGCCTCCT
	R: TGAGTCCTTCCACGATACCAA
U6	F: CTCGCTTCGGCAGCACA
	R: AACGCTTCACGAATTTGCGT

### Western blotting

Total protein extraction and quantification were performed using a total protein extraction kit (KeyGEN BioTECH) and a BCA protein content detection kit (KeyGEN BioTECH), respectively, according to the manufacturer’s protocols. As described by previous study (Chehade et al., 2021), the protein samples were separated by sodium dodecyl sulfate-polyacrylamide gel electrophoresis (SDS-PAGE) and subsequently transferred to polyvinylidene fluoride (PVDF) membranes. Immunoblotting was conducted using primary antibodies against SERPINE1 (ab222754, Abcam), E-cadherin (ab76055, Abcam), N-cadherin (66219-1-Ig, Proteintech), vimentin (bsm-33170m, Bioss), and GAPDH (ab9485, Abcam) at dilutions of 1:1000, 1:1000, 1:2000, 1:1000, and 1:2000, respectively. A secondary anti-rabbit IgG H&L antibody (ab6721, Abcam) was used at a dilution of 1:5000. Protein bands were visualized using an enhanced chemiluminescence (ECL) detection kit (KeyGEN BioTECH) and a ChemiDoc Touch 1708370 imaging system (Bio-Rad, USA). Densitometric analysis was performed using ImageJ software.

### Dual-luciferase reporter assay

The 293T cells were seeded into 12-well plates. Upon reaching approximately 50% confluency, pmirGLO-SERPINE1-WT or pmirGLO-SERPINE1-MUT recombinant plasmid was co-transfected with miR-642a-3p mimics or negative control into 293T cells using Lipofectamine™ 3000 transfection reagent (Invitrogen, USA). After a 48-hour transfection period, 50 µL of cell lysate was transferred to each well of a 96-well black plate. Subsequently, Dual-Glo® Luciferase Reagent (Promega, USA) was added, and firefly luciferase luminescence was quantified using a Tecan Spark microplate reader (Tecan, Männedorf, Switzerland). Finally, 100 µL of Dual-Glo® Stop & Glo® Reagent (Promega, Madison, WI, USA) was added to each well to measure Renilla luciferase luminescence. Relative luciferase activity was normalized to Renilla luciferase activity.

### Animal model

Four- to six-week-old male BALB/c nude mice obtained from Shanghai Lingchang Biotechnology Co., Ltd. were housed in single cages. The study protocol was approved by the Institutional Animal Care and Use Committee of Nanjing Ramda Pharmaceutical Co., Ltd. (IACUC-20230505) and carried out in accordance with the guidelines of the Animal Care Committee. Mice were acclimatized for one week under standard specific pathogen-free (SPF) conditions with a temperature of 20–26 °C, relative humidity of 40–70%, and a 12-hour light/12-hour dark cycle. Ten mice were randomly assigned to two groups: NC and shmiR-642a-3p (*n* = 5). Under abdominal anesthesia, nude mice were positioned supine, and the surgical site was disinfected. A midline abdominal incision was made to expose the liver, and the liver lobe exterior to the incision was gently removed with a cotton swab. Tumor cells were injected into the liver parenchyma approximately three mm deep using a needle, delivering 100 µL of Huh7-luc cells (Shanghai Zhong Qiao Xin Zhou Biotechnology Co., Ltd., China) at a density of 2 × 10^8^ cells/mL. The liver was repositioned, and the abdomen was closed layer by layer. One week post-inoculation, mice in the NC and shmiR-642a-3p groups received intraperitoneal injections of 2 × 10^11^ vg of AAV-vector or AAV-shmiR-642a-3p, respectively. The animals (*n* = 5 per group) were monitored twice weekly for behavioral changes, food consumption, and weight. At eight weeks, one mouse from each group was anesthetized with carbon dioxide and subjected to liver tumor nodule examination (Fig. S1). The remaining mice were euthanized with carbon dioxide, and their livers were excised and photographed. Liver tissues were divided: one half was fixed in 4% paraformaldehyde, while the other half was snap-frozen in liquid nitrogen and stored at −80 °C.

### Hematoxylin-eosin (H & E) staining

Liver tissues were fixed in a 4% paraformaldehyde solution and subsequently embedded in paraffin. Tissue sections with a thickness of 4 µm were prepared and stained with H&E. Histomorphological analysis of each liver was conducted using a SLIDEVIEW VS200 research slide scanner (Olympus).

### Immunohistochemistry (IHC) assay

Livers were immediately immersed in 4% paraformaldehyde solution and fixed overnight. Tissue blocks were embedded in paraffin and sectioned to a thickness of 4 µm. Immunohistochemical staining for Ki67 expression in liver tissue was performed using the EnVision two-step method. Rabbit anti-Ki67 (ab16667; Abcam) at a dilution of 1:50 served as the primary antibody. Subsequently, sections were incubated with a specified HRP-conjugated secondary antibody (MXB Biotechnologies, Fuzhou, China). Diaminobenzidine (DAB) solution (MXB Biotechnologies) and hematoxylin (Nanjing Jiancheng Bioengineering Institute, Nanjing, China) were used for color development. Ki67 expression in liver tissue was visualized using a SLIDEVIEW VS200 research slide scanner (Olympus).

### Statistical analysis

Data were analyzed using SPSS version 21.0 and presented as mean ±  standard error (SE) with at least three times. Independent-sample *t*-tests were employed to compare differences between two groups, while one-way analysis of variance (ANOVA) was used to assess differences among multiple groups. Post hoc comparisons were conducted using the Tukey method. All experiments were repeated at least three times. A *P*-value less than 0.05 was considered statistically significant.

## Results

### CAF-induced *SERPINE1* underexpression promoted proliferation and invasion of Huh7 cells

**Figure 1:**
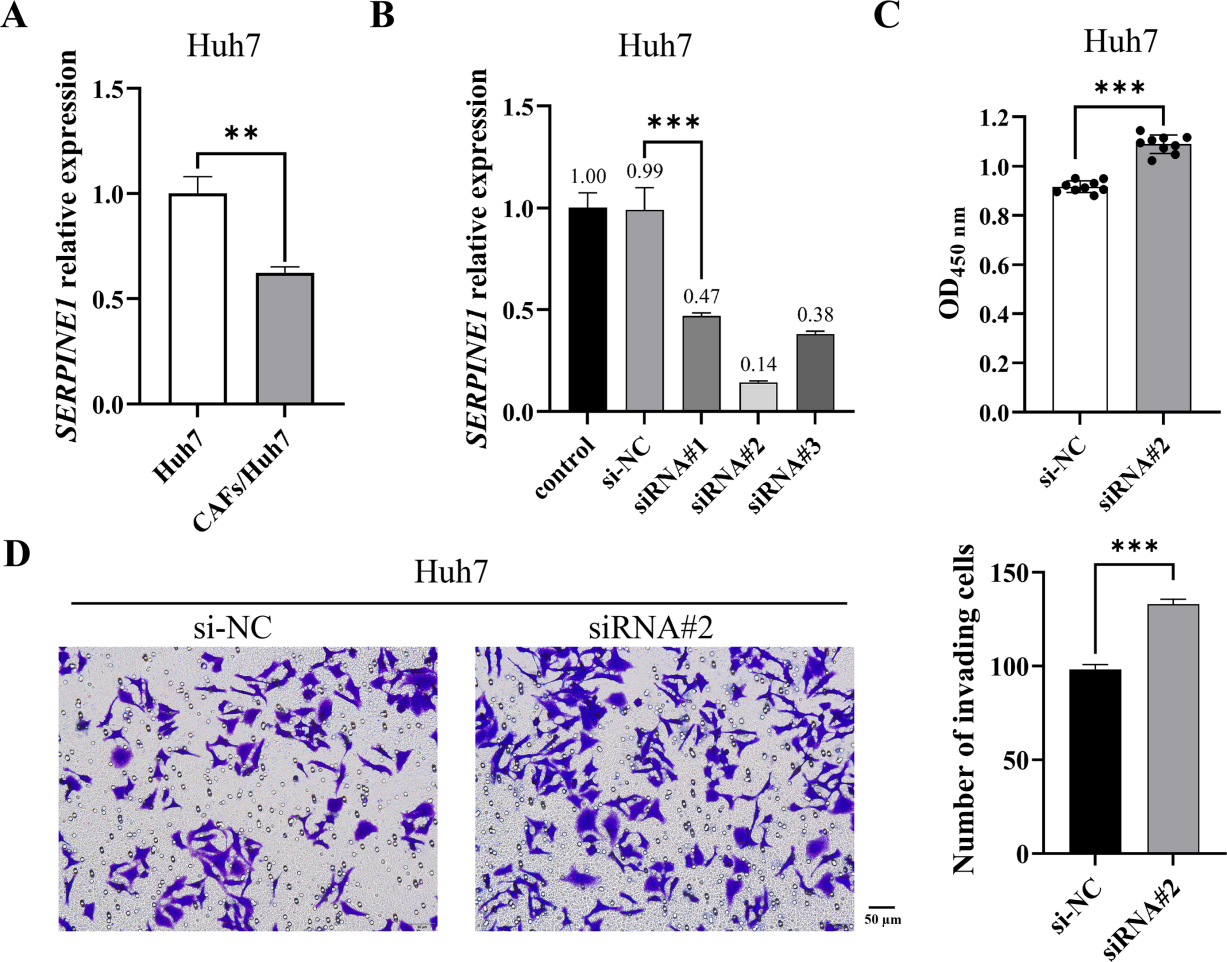
CAF-induced *SERPINE1* knockdown promoted Huh7 cell proliferation and invasion. (A) *SERPINE1* mRNA expression in Huh7 cells was measured by RT-qPCR. (B) RT-qPCR was used to detect the expression of *SERPINE1* mRNA following 48 h of *SERPINE1* siRNA transfection. (C) The proliferation of Huh7 cells was detected by CCK-8 assay. (D) The invasion of Huh7 cells was detected by the transwell chamber. Magnification: 20×. Data are presented as the mean ± standard error (*n* = 3). *P < 0.05, **P < 0.01, and ***P < 0.001.

CAFs are pivotal contributors to tumor progression, exhibiting a diverse range of functions encompassing collagen deposition and immunosuppression. Our prior investigation revealed that CAFs stimulated the proliferation and migration of Huh7 cells. To elucidate whether CAFs regulate Huh7 cells *via SERPINE1*, we initially quantified *SERPINE1* mRNA expression in Huh7 cells co-cultured with CAFs. The results indicated a significant decrease in *SERPINE1* mRNA expression within the co-culture group relative to the control group (Fig. 1A). Subsequently, *SERPINE1* gene knockdown markedly enhanced Huh7 cell proliferation and invasion (*P* < 0.001) (Figs. 1B–1D), suggesting that CAFs may promote Huh7 cell proliferation and invasion through the suppression of *SERPINE1* expression.

### CAFs inhibited *SERPINE1* expression in Huh7 cells by secreting miR-642a-3p

**Figure 2:**
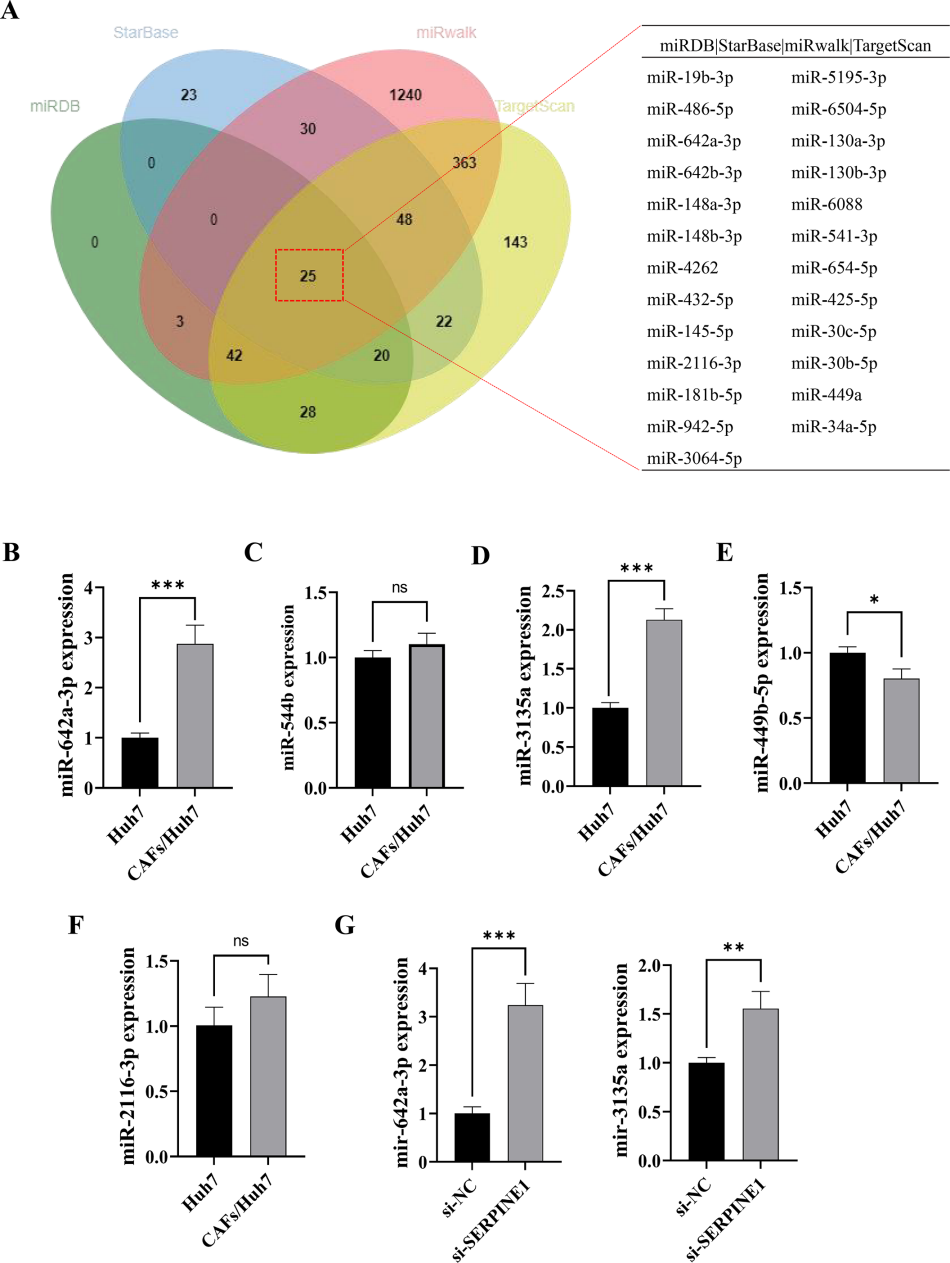
MiR-642a-3p might bind to SERPINE1. (A) Putative miRNAs binding to *SERPINE1* 3′ UTR using multiple miRNA databases, including StarBase, miRwalk, TargetScan, and miRDB. (B–F) Huh7 cells and CAFs were co-cultured in the transwell system for 48 hours, and the miRNA expression levels were measured by RT-qPCR. (G) MiR-642a-3p and miR-3135a expression in Huh7 cells following 48 h of *SERPINE1* siRNA transfection. Data are presented as the mean ± standard error (*n* = 3). *P < 0.05, **P < 0.01, and ***P < 0.001.

MicroRNAs function as intercellular messengers, transmitting information between cells, tissues, and organs. Within the TME, miRNAs contribute to tumor initiation and progression by regulating aberrant gene expression. To investigate whether CAFs influence *SERPINE1* expression in Huh7 cells through miRNA secretion, we identified 18 potential miRNAs targeting the *SERPINE1* 3′ UTR through multiple miRNA online databases (Fig. 2A). Subsequent screening revealed five miRNAs of interest, among which miR-642a-3p, miR-3135a, and miR-449b-5p exhibited significantly elevated expression in the co-culture group (*P* < 0.05) (Figs. 2B–2F). Moreover, miR-642a-3p and miR-3135a expression was markedly increased in Huh7 cells with *SERPINE1* knockdown (*P* < 0.01), with miR-642a-3p demonstrating a more pronounced difference between groups (Fig. 2G). These findings collectively suggest that CAFs suppress *SERPINE1* expression in Huh7 cells by secreting miR-642a-3p.

### CAFs-derived miR-642a-3p promoted migration, invasion, and EMT of Huh7 cells by inhibiting SERPINE1

**Figure 3:**
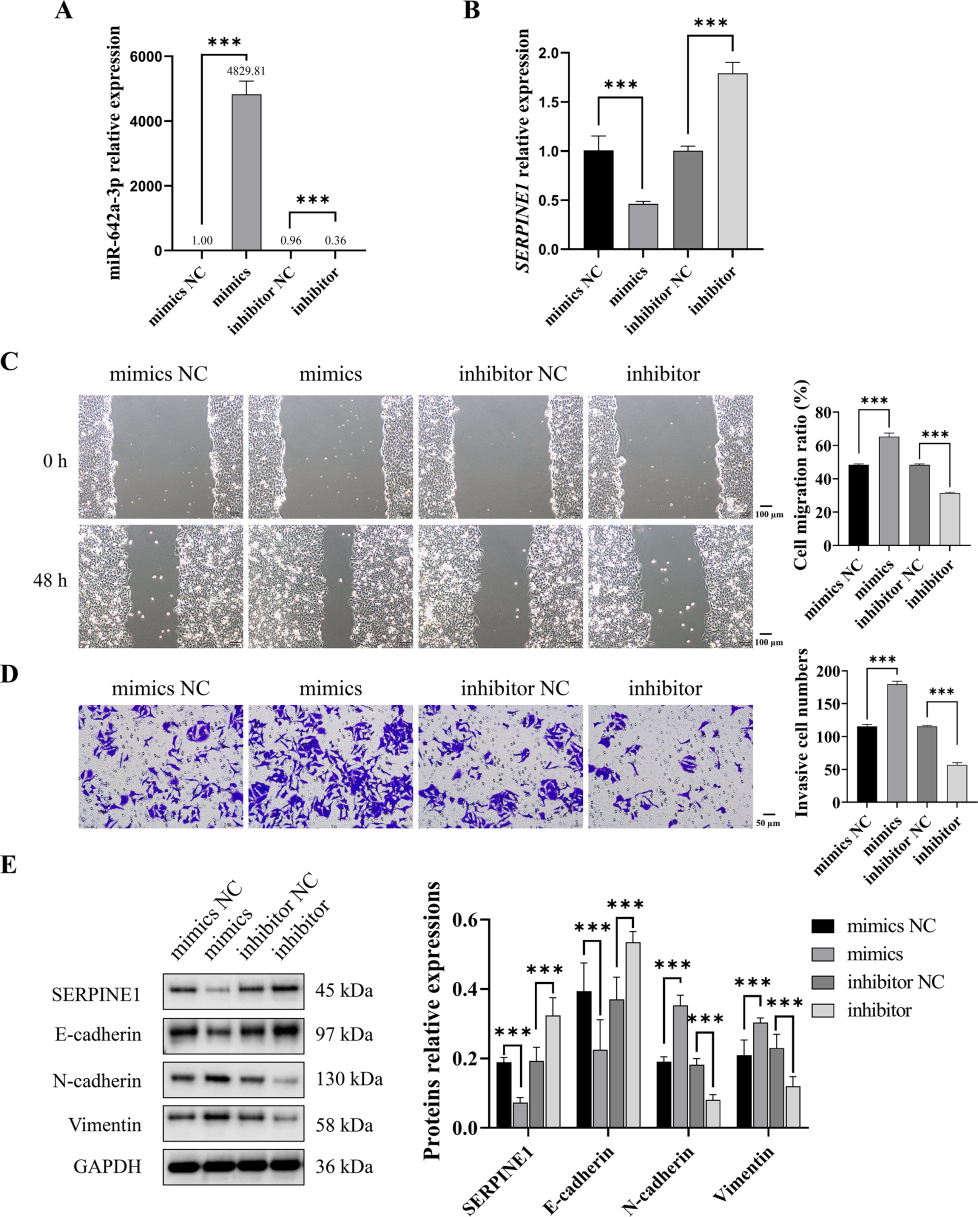
MiR-642a-3p promoted migration, invasion, and EMT of Huh7 cells. (A–B) After 48 h of miR-642a-3p mimics or inhibitor transfection, miR-642a-3p and *SERPINE1* mRNA expression in Huh7 cells were detected using RT-qPCR. (C) Cell migration ratio was measured by wound healing assay. Magnification: 10×. (D) Huh7 cell invasion was detected by transwell assay. Magnification: 20×. (E) Protein expression was examined by western blot. Data are presented as the mean ± standard error (*n* = 3). ns: *P* > 0.05, *P < 0.05, **P < 0.01, and ***P < 0.001.

To investigate whether CAF-derived miR-642a-3p promotes Huh7 cell migration, invasion, and EMT by regulating SERPINE1, miR-642a-3p mimics or inhibitors were transfected into Huh7 cells. Results demonstrated that miR-642a-3p mimics significantly upregulated miR-642a-3p expression while downregulating *SERPINE1* mRNA expression (*P* < 0.001), whereas the miR-642a-3p inhibitor exhibited the opposite effect (Figs. 3A and 3B). Moreover, miR-642a-3p mimics significantly enhanced Huh7 cell migration and invasion, while the miR-642a-3p inhibitor significantly suppressed these processes (Figs. 3C and 3D). Notably, miR-642a-3p mimics markedly increased N-cadherin and vimentin protein expression and decreased E-cadherin protein expression in Huh7 cells (*P* < 0.001) (Fig. 3E). Conversely, the miR-642a-3p inhibitor significantly upregulated E-cadherin protein expression and downregulated N-cadherin and vimentin protein expression (*P* < 0.001) (Fig. 3E), suggesting that CAF-derived miR-642a-3p promotes EMT in Huh7 cells.

**Figure 4:**
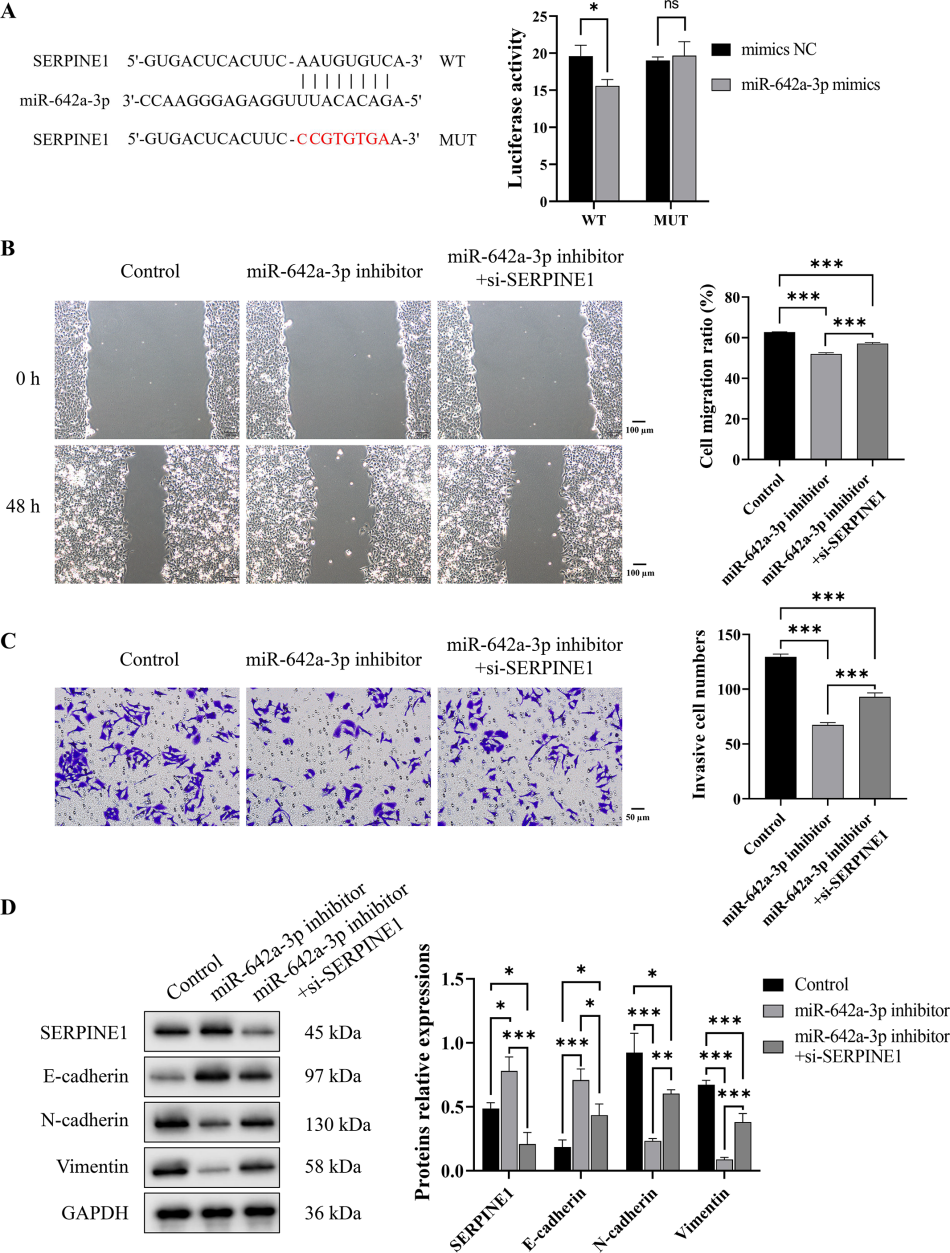
MiR-642a-3p knockdown inhibited migration, invasion, and EMT of Huh7 cells by targeting SERPINE1. (A) The binding of miR-642a-3p with *SERPINE1* 3′ UTR was detected by dual-Luciferase reporter assay. (B) After miR-642a-3p inhibitor or miR-642a-3p inhibitor combined with *SERPINE1* siRNA was transfected into Huh7 cells for 48 h, the cell migration ratio was measured by wound healing assay. Magnification: 10×. (C) Huh7 cell invasion was detected by transwell assay. Magnification: 20×. (D) Protein expression was examined by western blot. Data are presented as the mean ± standard error (*n* = 3). ns: *P* > 0.05, *P < 0.05, **P < 0.01, and ***P < 0.001.

To investigate the binding interaction between miR-642a-3p and the 3′ UTR of *SERPINE1*, recombinant plasmids pmirGLO-SERPINE1-WT and -MUT were constructed based on predicted binding sites from a miRNA database. A dual-luciferase reporter assay demonstrated a significant decrease in fluorescence activity in the WT group upon miR-642a-3p mimic overexpression (*P* < 0.05), whereas no effect was observed in the MUT group (*P* > 0.05) (Fig. 4A). Rescue experiments revealed that *SERPINE1* knockdown attenuated the inhibitory effects of miR-642a-3p knockdown on cell migration, invasion, and EMT in Huh7 cells (Figs. 4B–4D), suggesting that miR-642a-3p promotes migration, invasion, and EMT in Huh7 cells by targeting SERPINE1.

### miR-642a-3p knockdown inhibited tumor growth and EMT *in vivo*

**Figure 5:**
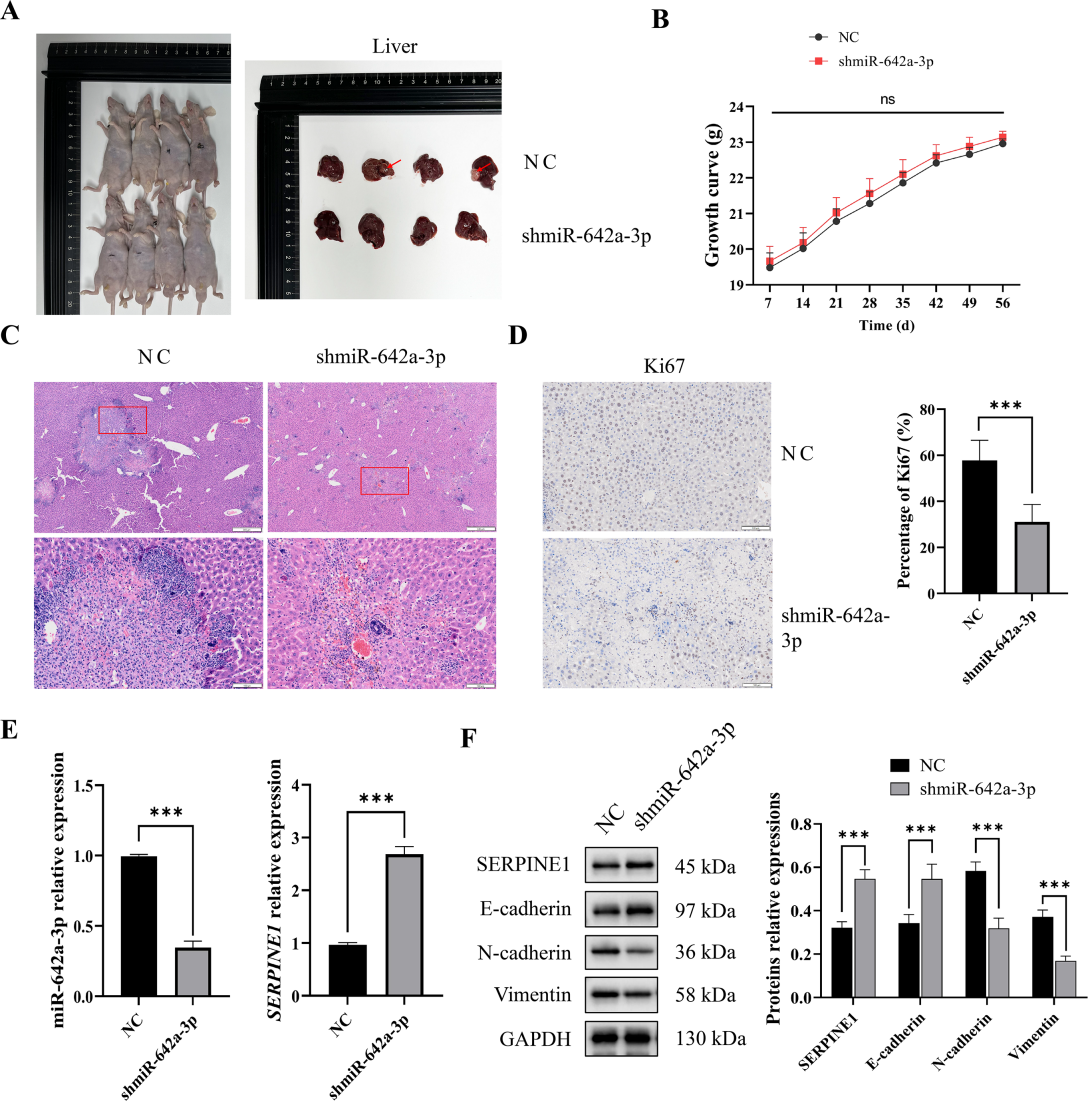
MiR-642a-3p knockdown suppressed growth of orthotopic liver tumors. (A) Nude mice and liver tissues in the NC and shmiR-642a-3p groups. Four animals were shown in each group (*n* = 5). (B) Growth curve of the nude mice. (C) The structures of the liver tissues were observed by HE staining (*n* = 3). Magnification: 2× and 10×. (D) Ki67 expressions were analyzed using immunohistochemistry (*n* = 3). Magnification: 10×. (E) Expression levels of miR-642a-3p and *SERPINE1* gene were detected by RT-qPCR (*n* = 3). (F) Protein expression was analyzed using western blot (*n* = 5). *P < 0.05, **P < 0.01, and ***P < 0.001.

To assess the *in vivo* impact of miR-642a-3p on tumor infiltration, an orthotopic tumor model was established by injecting Huh7 cells into the livers of nude mice. As depicted in Fig. 5A, tumors were observed in the NC group but absent in the shmiR-642a-3p group. While no significant weight difference was observed between groups, a slight increase in weight was noted for the shmiR-642a-3p group (Fig. 5B). Histopathological examination of the NC group revealed visible tumor lesions invading the hepatic parenchyma with a larger invasion area compared to the reduced invasion area observed in the shmiR-642a-3p group (Figs. 5C and 5D). Moreover, miR-642a-3p knockdown significantly suppressed miR-642a-3p expression while enhancing *SERPINE1* expression (Fig. 5E). Notably, miR-642a-3p knockdown upregulated SERPINE1 and E-cadherin protein levels while downregulating N-cadherin and vimentin protein levels (Fig. 5F), indicating an inhibitory effect on tumor infiltration and dissemination.

## Discussion

The TME is a critical determinant of cancer initiation and progression (Ye et al., 2024; Yu, Huang & Guo, 2024). In the TME, a large number of CAFs are recruited and activated, thus affecting cancer progression (Jing et al., 2024; Wang et al., 2024). It has been reported that CAFs are activated, proliferate, and accumulate in over 80% of HCC cases (Affo, Yu & Schwabe, 2017). These activated CAFs exert carcinogenic effects through multiple mechanisms, including the secretion of soluble factors and exosomes, as well as extracellular matrix (ECM) remodeling (Yin et al., 2019; Ying, Chan & Lee, 2023). Our findings revealed a significant decrease in *SERPINE1* mRNA expression in Huh7 cells co-cultured with CAFs. Subsequent investigations demonstrated that *SERPINE1* knockdown markedly enhanced Huh7 cell proliferation and invasion. This suggests that CAFs may accelerate the development of HCC by causing *SERPINE1* gene to express poorly in HCC cells.

The role of SERPINE1 in cancer remains controversial (Jin et al., 2020; Nam, Seong & Hahn, 2021; Zhu et al., 2020). In gastric cancer, SERPINE1 knockdown significantly inhibited cell proliferation, migration, invasion, and xenograft tumor growth (Chen et al., 2022). SERPINE1 has been directly linked to EMT, tumor cell stemness, and chemoresistance in head and neck squamous cell carcinoma, with its overexpression correlating with increased metastasis risk (Pavón et al., 2016). Interestingly, SERPINE1 has been shown to promote senescence in lung cancer cells (A549 and H1299), thereby inhibiting tumor progression (Wang et al., 2023a). However, conflicting findings indicate that SERPINE1 upregulation promotes lung cancer cell invasion (Kong et al., 2021). Within the context of HCC, SERPINE1 is predominantly considered an oncogene, although some studies suggest an anti-cancer role. Jin et al., (2020) reported significantly higher SERPINE1 expression in HCC tissues compared to adjacent non-cancerous tissues, with a negative correlation between SERPINE1 expression and overall survival, suggesting its prognostic value (Jin et al., 2020). SERPINE1 has been shown to promote proliferation, migration and invasion in HepG2 cells (Li et al., 2021b). Conversely, our findings demonstrate that *SERPINE1* knockdown enhances proliferation and invasion in Huh7 cells, indicating the heterogeneous nature of SERPINE1 expression and function within HCC cells. Notably, CAF-derived SERPINE1 exhibits tumor-suppressor activity in Huh7 cells.

MiRNAs are single-stranded, non-coding RNAs. They regulate gene expression by binding to the 3′ UTR of target messenger RNAs (mRNAs), leading to mRNA degradation or translational inhibition (Ali Syeda et al., 2020; Diener, Keller & Meese, 2024). Each miRNA can regulate multiple target genes, influencing a diverse array of biological processes, including differentiation, development, proliferation, migration, and apoptosis (Ali Syeda et al., 2020; Nemeth et al., 2024). In recent years, miRNAs have emerged as promising biomarkers for tumor diagnosis (Calis, Mogulkoc & Baltaci, 2020; He et al., 2020; Li et al., 2021a). Dysregulation of miRNAs, such as miR-155 (Vo et al., 2012), miR-541 (Xu et al., 2020), miR-126 (Zailaie & Sergi, 2022), and miR-17-5p (Zhou et al., 2024), has been implicated in the malignant progression and poor prognosis of HCC. These miRNAs influence critical HCC processes, including proliferation, apoptosis, metastasis, and drug resistance (Mallela et al., 2024). Moreover, miRNAs function as signaling molecules facilitating intercellular communication, enabling information exchange and gene regulation between tumor cells and other cell types, including CAFs and immune cells, ultimately contributing to tumor progression (Barrera et al., 2023; Qi et al., 2022; Salah et al., 2022). For instance, CAF-derived exosomal miR-20a-5p promotes HCC progression through the LIMA1-mediated *β*-catenin pathway (Qi et al., 2022), while CAF-derived exosomal miR-1228-3p enhances the resistance of liver cancer cells to sorafenib (Zhang, Pan & Shao, 2023).

Our study revealed that miR-642a-3p expression increased concurrently with a decrease in *SERPINE1* expression in Huh7 cells co-cultured with CAFs, suggesting a potential inhibitory effect of CAFs on *SERPINE1* expression in Huh7 cells *via* secreting miR-642a-3p. While limited research has explored miR-642a-3p in cancer, existing studies indicate its role in promoting tumor invasion, metastasis (Cao et al., 2022), and drug resistance (Qin et al., 2017; Yu et al., 2019). To investigate the involvement of CAF-derived miR-642a-3p in HCC progression through targeting SERPINE1, we confirmed the binding of miR-642a-3p to the *SERPINE1* 3′ UTR using a dual-luciferase reporter assay. Subsequent *in vitro* studies demonstrated that CAF-derived miR-642a-3p promotes HCC cell migration, invasion, and EMT by targeting SERPINE1. Moreover, *in vivo* experiments revealed that miR-642a-3p knockdown significantly suppressed tumor proliferation and dissemination in the liver, highlighting its critical role in HCC progression. Given the established role of exosomes in miRNA-mediated intercellular communication (Sheng et al., 2024; Zhang et al., 2024), future investigations will focus on determining whether CAF-derived miR-642a-3p is encapsulated in CAF-secreted exosomes and elucidating the functional implications of exosomal miR-642a-3p in CAF-HCC crosstalk.

At present, our research still has some limitations. While our study confirms that *SERPINE1* knockdown promotes proliferation of the HCC cells, we did not explore the effect of CAF-derived miR-642a-3p/SERPINE1 axis on the cell proliferation, necrosis, or apoptosis, which is the most direct demonstration of the effect on tumors. Next, we will evaluate the role of miR-642a-3p/SERPINE1 axis in HCC cell proliferation, necrosis, and apoptosis using CCK-8, LDH cytotoxicity, and Annexin V/PI apoptosis assays. In addition, we will examine the expression of miR-642a-3p and SERPINE1 to assess their clinical implications for HCC staging and prognosis, using HCC tissue microarray technology. More significantly, using transcriptome, proteome, and metabolome sequencings, we will investigate the molecular mechanism of miR-642a-3p/SERPINE1 axis in multiple HCC cell lines to provide new insights for diagnosis and treatment of HCC.

**Figure 6:**
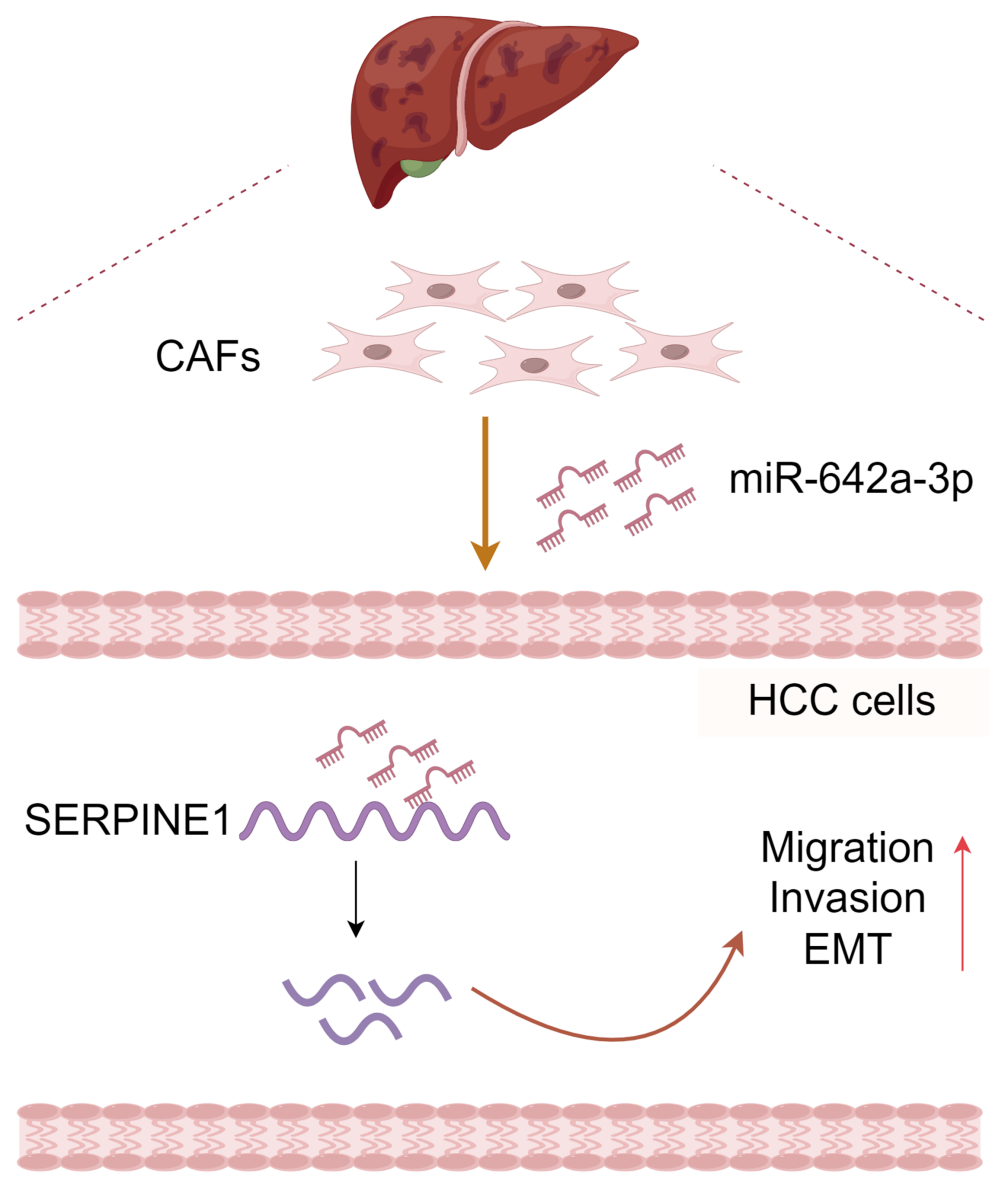
Molecular pattern of CAFs-drived miR-642a-3p supporting the migration, invasion, and EMT of hepatocellular carcinoma by targeting SERPINE1. Created with Figdraw.

In conclusion, CAF-derived miR-642a-3p promotes hepatocellular carcinoma cell migration, invasion, and EMT by targeting SERPINE1 (Fig. 6), suggesting its potential as a molecular marker for HCC treatment. Additionally, our study enriches the intricate functions of SERPINE1 in HCC.

##  Supplemental Information

10.7717/peerj.18428/supp-1 Supplemental Information 1 The ARRIVE guidelines 2.0: author checklist

10.7717/peerj.18428/supp-2 Supplemental Information 2 MIQE checklist

10.7717/peerj.18428/supp-3 Supplemental Information 3 RT-qPCR analysis of SERPINE1 mRNA levels in Huh7 cells after coculture with CAFs

10.7717/peerj.18428/supp-4 Supplemental Information 4 RT-qPCR analysis of SERPINE1 mRNA levels in Huh7 cells after SERPINE1 siRNA

10.7717/peerj.18428/supp-5 Supplemental Information 5 RT-qPCR analysis of miR-642a-3p, miR-3135a, miR-449b-5p, miR-2116-3p, and miR-544b levels in Huh7 cells after coculture with CAFs

10.7717/peerj.18428/supp-6 Supplemental Information 6 RT-qPCR analysis of miR-3135a and miR-642a-3p levels in Huh7 cells after SERPINE1 siRNA

10.7717/peerj.18428/supp-7 Supplemental Information 7 RT-qPCR analysis of miR-642a-3p and SERPINE1 mRNA levels in Huh7 cells after miR-642a-3p mimics or inhibitor

10.7717/peerj.18428/supp-8 Supplemental Information 8 The full-length uncropped gels/blots of SERPINE1 and EMT-related protein expressions in Huh7 cells after miR-642a-3p mimics or inhibitor

10.7717/peerj.18428/supp-9 Supplemental Information 9 The full-length uncropped gels/blots of SERPINE1 and EMT-related protein expressions in Huh7 cells after miR-642a-3p inhibitor or miR-642a-3p inhibitor combined with si-SERPINE1

10.7717/peerj.18428/supp-10 Supplemental Information 10 Nude mice and liver tissues in the NC and shmiR-642a-3p groups

10.7717/peerj.18428/supp-11 Supplemental Information 11 RT-qPCR analysis of miR-642a-3p and SERPINE1 mRNA levels after miR-642a-3p knockdown

10.7717/peerj.18428/supp-12 Supplemental Information 12 The full-length uncropped gels/blots of SERPINE1 and EMT-related protein expressions after miR-642a-3p knockdown

10.7717/peerj.18428/supp-13 Supplemental Information 13 Effect of miR-642a-3p knockdown on growth of orthotopic liver tumors One animal were shown first in each group (*n* = 5).

## Abbreviations

CAFs: cancer-associated fibroblasts
HCC: hepatocellular carcinoma
SERPINE1: serpin family E member 1
ncRNAs: non-coding RNAs
miRNAs: microRNAs
CCK-8: Cell Counting Kit-8
RT-qPCR: real-time quantitative polymerase chain reaction
EMT: epithelial-mesenchymal transition
NASH: non-alcoholic steatohepatitis
HBV: hepatitis B virus
HCV: hepatitis C virus
TME: tumor microenvironment
TAMs: tumor-associated macrophages
PVDF: polyvinylidene fluoride
ANOVA: one-way analysis of variance
SE: standard error
ECM: extracellular matrix
UTR: untranslated region

## Additional Information and Declarations

### Data Availability

The following information was supplied regarding data availability: The raw data are available in the Supplementary Files.

## References

[ref-1] Affo S, Yu LX, Schwabe RF (2017). The role of cancer-associated fibroblasts and fibrosis in liver cancer. Annual Review of Pathology.

[ref-2] Ali Syeda Z, Langden SSS, Munkhzul C, Lee M, Song SJ (2020). Regulatory mechanism of MicroRNA expression in cancer. International Journal of Molecular Sciences.

[ref-3] Arpinati L, Carradori G, Scherz-Shouval R (2024). CAF-induced physical constraints controlling T cell state and localization in solid tumours. Nature Reviews Cancer.

[ref-4] Barrera LN, Ridley PM, Bermejo-Rodriguez C, Costello E, Perez-Mancera PA (2023). The role of microRNAs in the modulation of cancer-associated fibroblasts activity during pancreatic cancer pathogenesis. Journal of Physiology and Biochemistry.

[ref-5] Basak U, Sarkar T, Mukherjee S, Chakraborty S, Dutta A, Dutta S, Nayak D, Kaushik S, Das T, Sa G (2023). Tumor-associated macrophages: an effective player of the tumor microenvironment. Frontiers in Immunology.

[ref-6] Biffi G, Tuveson DA (2021). Diversity and biology of cancer-associated fibroblasts. Physiological Reviews.

[ref-7] Caligiuri G, Tuveson DA (2023). Activated fibroblasts in cancer: perspectives and challenges. Cancer Cell.

[ref-8] Calis Z, Mogulkoc R, Baltaci AK (2020). The roles of flavonols/flavonoids in neurodegeneration and neuroinflammation. Mini-Reviews in Medicinal Chemistry.

[ref-9] Cao J, Shao H, Hu J, Jin R, Feng A, Zhang B, Li S, Chen T, Jeungpanich S, Topatana W, Tian Y, Lu Z, Cai X, Chen M (2022). Identification of invasion-metastasis associated MiRNAs in gallbladder cancer by bioinformatics and experimental validation. Journal of Translational Medicine.

[ref-10] Chakrabortty A, Patton DJ, Smith BF, Agarwal P (2023). miRNAs: potential as biomarkers and therapeutic targets for cancer. Genes.

[ref-11] Chehade H, Fox A, Mor GG, Alvero AB (2021). Determination of caspase activation by western blot. Methods in Molecular Biology.

[ref-12] Chen S, Li Y, Zhu Y, Fei J, Song L, Sun G, Guo L, Li X (2022). SERPINE1 overexpression promotes malignant progression and poor prognosis of gastric cancer. Journal of Oncology.

[ref-13] Chen S, Morine Y, Tokuda K, Yamada S, Saito Y, Nishi M, Ikemoto T, Shimada M (2021). Cancer-associated fibroblast-induced M2-polarized macrophages promote hepatocellular carcinoma progression *via* the plasminogen activator inhibitor-1 pathway. International Journal of Oncology.

[ref-14] Diener C, Keller A, Meese E (2024). The miRNA-target interactions: an underestimated intricacy. Nucleic Acids Research.

[ref-15] Ganesan P, Kulik LM (2023). Hepatocellular carcinoma: new developments. Clinical Liver Disease.

[ref-16] Ghafouri-Fard S, Honarmand Tamizkar K, Hussen BM, Taheri M (2021). MicroRNA signature in liver cancer. Pathology—Research and Practice.

[ref-17] He B, Zhao Z, Cai Q, Zhang Y, Zhang P, Shi S, Xie H, Peng X, Yin W, Tao Y, Wang X (2020). miRNA-based biomarkers, therapies, and resistance in cancer. International Journal of Biological Sciences.

[ref-18] Helms E, Onate MK, Sherman MH (2020). Fibroblast heterogeneity in the pancreatic tumor microenvironment. Cancer Discovery.

[ref-19] Higgins CE, Tang J, Mian BM, Higgins SP, Gifford CC, Conti DJ, Meldrum KK, Samarakoon R, Higgins PJ (2019). TGF- *β*1-p53 cooperativity regulates a profibrotic genomic program in the kidney: molecular mechanisms and clinical implications. The FASEB Journal.

[ref-20] Hoekstra ME, Slagter M, Urbanus J, Toebes M, Slingerland N, De Rink I, Kluin RJC, Nieuwland M, Kerkhoven R, Wessels LFA, Schumacher TN (2024). Distinct spatiotemporal dynamics of CD8(+) T cell-derived cytokines in the tumor microenvironment. Cancer Cell.

[ref-21] Hong CL, Yu IS, Pai CH, Chen JS, Hsieh MS, Wu HL, Lin SW, Huang HP (2022). CD248 regulates wnt signaling in pericytes to promote angiogenesis and tumor growth in lung cancer. Cancer Research.

[ref-22] Hou Z, Liu J, Jin Z, Qiu G, Xie Q, Mi S, Huang J (2022). Use of chemotherapy to treat hepatocellular carcinoma. BioScience Trends.

[ref-23] Hu Y, Setayesh T, Vaziri F, Wu X, Hwang ST, Chen X, Wan YJY (2023). miR-22 gene therapy treats HCC by promoting anti-tumor immunity and enhancing metabolism. Molecular Therapy.

[ref-24] Iaccarino I, Klapper W (2021). LncRNA as cancer biomarkers. Methods in Molecular Biology.

[ref-25] Jin Y, Liang ZY, Zhou WX, Zhou L (2020). Expression, clinicopathologic and prognostic significance of plasminogen activator inhibitor 1 in hepatocellular carcinoma. Cancer Biomarkers.

[ref-26] Jing SY, Liu D, Feng N, Dong H, Wang HQ, Yan X, Chen XF, Qu MC, Lin P, Yi B, Feng F, Chen L, Wang HY, Li H, He YF (2024). Spatial multiomics reveals a subpopulation of fibroblasts associated with cancer stemness in human hepatocellular carcinoma. Genome Medicine.

[ref-27] Ju Y, Wang Z, Wang Q, Jin S, Sun P, Wei Y, Zhu G, Wang K (2024). Pan-cancer analysis of SERPINE1 with a concentration on immune therapeutic and prognostic in gastric cancer. Journal of Cellular and Molecular Medicine.

[ref-28] Kong HJ, Kwon EJ, Kwon OS, Lee H, Choi JY, Kim YJ, Kim W, Cha HJ (2021). Crosstalk between YAP and TGF *β* regulates SERPINE1 expression in mesenchymal lung cancer cells. International Journal of Oncology.

[ref-29] Ladd AD, Duarte S, Sahin I, Zarrinpar A (2024). Mechanisms of drug resistance in HCC. Hepatology.

[ref-30] Li B, Cao Y, Sun M, Feng H (2021a). Expression, regulation, and function of exosome-derived miRNAs in cancer progression and therapy. The FASEB Journal.

[ref-31] Li LM, Chen C, Ran RX, Huang JT, Sun HL, Zeng C, Zhang Z, Zhang W, Liu SM (2021b). Loss of TARBP2 drives the progression of hepatocellular carcinoma *via* miR-145-SERPINE1 axis. Frontiers in Oncology.

[ref-32] Lv Y, Sun X (2024). Role of miRNA in pathogenesis, diagnosis, and prognosis in hepatocellular carcinoma. Chemical Biology & Drug Design.

[ref-33] Mallela VR, Rajtmajerová M, Trailin A, Liška V, Hemminki K, Ambrozkiewicz F (2024). miRNA and lncRNA as potential tissue biomarkers in hepatocellular carcinoma. Non-coding RNA Research.

[ref-34] Miyai Y, Esaki N, Takahashi M, Enomoto A (2020). Cancer-associated fibroblasts that restrain cancer progression: Hypotheses and perspectives. Cancer Science.

[ref-35] Nagy Á, Munkácsy G, Győrffy B (2021). Pancancer survival analysis of cancer hallmark genes. Scientific Reports.

[ref-36] Nam DE, Seong HC, Hahn YS (2021). Plasminogen activator inhibitor-1 and oncogenesis in the liver disease. Journal of Cellular Signaling.

[ref-37] Nemeth K, Bayraktar R, Ferracin M, Calin GA (2024). Non-coding RNAs in disease: from mechanisms to therapeutics. Nature Reviews Genetics.

[ref-38] Parsa-Kondelaji M, Musavi M, Barzegar F, Abbasian N, Rostami M, RS M, SH S, Modi M, Nikfar B, Momtazi-Borojeni A (2023). Dysregulation of miRNA expression in patients with chronic myelogenous leukemia at diagnosis: a systematic review. Biomarkers in Medicine.

[ref-39] Pavón MA, Arroyo-Solera I, Céspedes MV, Casanova I, León X, Mangues R (2016). uPA/uPAR and SERPINE1 in head and neck cancer: role in tumor resistance, metastasis, prognosis and therapy. Oncotarget.

[ref-40] Phoolchund AGS, Khakoo SI (2024). MASLD and the development of HCC: pathogenesis and therapeutic challenges. Cancers.

[ref-41] Qi Y, Wang H, Zhang Q, Liu Z, Wang T, Wu Z, Wu W (2022). CAF-released exosomal miR-20a-5p facilitates HCC progression *via* the LIMA1-mediated *β*-catenin pathway. Cells.

[ref-42] Qin X, Yu S, Xu X, Shen B, Feng J (2017). Comparative analysis of microRNA expression profiles between A549, A549/DDP and their respective exosomes. Oncotarget.

[ref-43] Rumgay H, Arnold M, Ferlay J, Lesi O, Cabasag CJ, Vignat J, Laversanne M, McGlynn KA, Soerjomataram I (2022). Global burden of primary liver cancer in 2020 and predictions to 2040. Journal of Hepatology.

[ref-44] Salah RA, Nasr MA, El-Derby AM, Abd Elkodous M, Mohamed RH, El-Ekiaby N, Osama A, Elshenawy SE, Hamad MHM, Magdeldin S, Gabr MM, Abdelaziz AI, El-Badri NS (2022). Hepatocellular carcinoma cell line-microenvironment induced cancer-associated phenotype, genotype and functionality in mesenchymal stem cells. Life Sciences.

[ref-45] Sankar K, Gong J, Osipov A, Miles SA, Kosari K, Nissen NN, Hendifar AE, Koltsova EK, Yang JD (2024). Recent advances in the management of hepatocellular carcinoma. Clinical and Molecular Hepatology.

[ref-46] Schneider P, Zhang H, Simic L, Dai Z, Schrörs B, Akilli-Öztürk Ö, Lin J, Durak F, Schunke J, Bolduan V, Bogaert B, Schwiertz D, Schäfer G, Bros M, Grabbe S, Schattenberg JM, Raemdonck K, Koynov K, Diken M, Kaps L, Barz M (2024). Multicompartment polyion complex micelles based on triblock polypept(o)ides mediate efficient siRNA delivery to cancer-associated fibroblasts for antistromal therapy of hepatocellular carcinoma. Advanced Materials.

[ref-47] Setiawan L, Setiabudy R, Kresno SB, Sutandyo N, Syahruddin E, Jovianti F, Nadliroh S, Mubarika S, Setiabudy R, Siregar NC (2024). Circulating miR-10b, soluble urokinase-type plasminogen activator receptor, and plasminogen activator inhibitor-1 as predictors of non-small cell lung cancer progression and treatment response. Cancer Biomarkers.

[ref-48] Shang H, Lu L, Fan M, Lu Y, Shi X, Lu H (2024). Exosomal circHIF1A derived from hypoxic-induced carcinoma-associated fibroblasts promotes hepatocellular carcinoma cell malignant phenotypes and immune escape. International Immunopharmacology.

[ref-49] Sheng Z, Wang X, Ding X, Zheng Y, Guo A, Cui J, Ma J, Duan W, Dong H, Zhang H, Cui M, Su W, Zhang B (2024). Exosomal miRNA-92a derived from cancer-associated fibroblasts promote invasion and metastasis in breast cancer by regulating G3BP2. Cell Signaling.

[ref-50] Sillen M, Declerck PJ (2021). A narrative review on plasminogen activator Iinhibitor-1 and its (patho)physiological role: to target or not to target?. International Journal of Molecular Sciences.

[ref-51] Su X, Wang X, Lai J, Mao S, Li H (2024a). Unraveling a novel hippo-associated immunological prognostic signature: the contribution of SERPINE1 in facilitating colorectal cancer progression *via* the notch signaling pathway. Genomics.

[ref-52] Su Z, Lu C, Zhang F, Liu H, Li M, Qiao M, Zou X, Luo D, Li H, He M, Se H, Jing J, Wang X, Yang H, Yang H (2024b). Cancer-associated fibroblasts-secreted exosomal miR-92a-3p promotes tumor growth and stemness in hepatocellular carcinoma through activation of Wnt/ *β*-catenin signaling pathway by suppressing AXIN1. Journal of Cellular Physiology.

[ref-53] Szymanowska A, Rodriguez-Aguayo C, Lopez-Berestein G, Amero P (2023). Non-coding RNAs: foes or friends for targeting tumor microenvironment. Noncoding RNA.

[ref-54] Teng F, Zhang JX, Chen Y, Shen XD, Su C, Guo YJ, Wang PH, Shi CC, Lei M, Cao YO, Liu SQ (2021). LncRNA NKX2-1-AS1 promotes tumor progression and angiogenesis *via* upregulation of SERPINE1 expression and activation of the VEGFR-2 signaling pathway in gastric cancer. Molecular Oncology.

[ref-55] Tutunchi S, Akhavan S, Bereimipour A, Hossein Ghaderian SM (2021). Evaluation of important molecular pathways and candidate diagnostic biomarkers of noninvasive to invasive stages in gastric cancer by *in silico* analysis. Journal of Oncology.

[ref-56] Vo VA, Lee JW, Chang JE, Kim JY, Kim NH, Lee HJ, Kim SS, Chun W, Kwon YS (2012). Avicularin inhibits lipopolysaccharide-induced inflammatory response by suppressing ERK phosphorylation in RAW 264.7 macrophages. Biomolecules & Therapeutics.

[ref-57] Vogel A, Meyer T, Sapisochin G, Salem R, Saborowski A (2022). Hepatocellular carcinoma. The Lancet.

[ref-58] Wang H, Liang Y, Liu Z, Zhang R, Chao J, Wang M, Liu M, Qiao L, Xuan Z, Zhao H, Lu L (2024). POSTN(+) cancer-associated fibroblasts determine the efficacy of immunotherapy in hepatocellular carcinoma. The Journal for ImmunoTherapy of Cancer.

[ref-59] Wang Y, Wang J, Gao J, Ding M, Li H (2023b). The expression of SERPINE1 in colon cancer and its regulatory network and prognostic value. BMC Gastroenterology.

[ref-60] Wang X, Wang N, Li H, Liu M, Cao F, Yu X, Zhang J, Tan Y, Xiang L, Feng Y (2016). Up-regulation of PAI-1 and down-regulation of uPA are involved in suppression of invasiveness and motility of hepatocellular carcinoma cells by a natural compound berberine. International Journal of Molecular Sciences.

[ref-61] Wang L, Zhang X, Sheng J, Chen L, Zhi L, Zheng Q, Qi Y, Wang L, Zhang J, Zhao J, Wang Y, Liu SX, Sun MZ, Zhang W (2023a). RBM4 regulates cellular senescence *via* miR1244/SERPINE1 axis. Cell Death & Disease.

[ref-62] Xu WP, Liu JP, Feng JF, Zhu CP, Yang Y, Zhou WP, Ding J, Huang CK, Cui YL, Ding CH, Zhang X, Lu B, Xie WF (2020). miR-541 potentiates the response of human hepatocellular carcinoma to sorafenib treatment by inhibiting autophagy. Gut.

[ref-63] Yasuda T, Wang YA (2024). Gastric cancer immunosuppressive microenvironment heterogeneity: implications for therapy development. Trends in Cancer.

[ref-64] Ye J, Baer JM, Faget DV, Morikis VA, Ren Q, Melam A, Delgado AP, Luo X, Bagchi SM, Belle JI, Campos E, Friedman M, Veis DJ, Knudsen ES, Witkiewicz AK, Powers S, Longmore GD, DeNardo DG, Stewart SA (2024). Senescent CAFs Mediate Immunosuppression and Drive Breast Cancer Progression. Cancer Discovery.

[ref-65] Yin Z, Dong C, Jiang K, Xu Z, Li R, Guo K, Shao S, Wang L (2019). Heterogeneity of cancer-associated fibroblasts and roles in the progression, prognosis, and therapy of hepatocellular carcinoma. Journal of Hematology & Oncology.

[ref-66] Ying F, Chan MSM, Lee TKW (2023). Cancer-associated fibroblasts in hepatocellular carcinoma and cholangiocarcinoma. Cellular and Molecular Gastroenterology and Hepatology.

[ref-67] Yu C, Chen DQ, Liu HX, Li WB, Lu JW, Feng JF (2019). Rosmarinic acid reduces the resistance of gastric carcinoma cells to 5-fluorouracil by downregulating FOXO4-targeting miR-6785-5p. Biomedicine & Pharmacotherapy.

[ref-68] Yu L, Shen N, Shi Y, Shi X, Fu X, Li S, Zhu B, Yu W, Zhang Y (2022). Characterization of cancer-related fibroblasts (CAF) in hepatocellular carcinoma and construction of CAF-based risk signature based on single-cell RNA-seq and bulk RNA-seq data. Frontiers in Immunology.

[ref-69] Yu Z, Huang L, Guo J (2024). Anti-stromal nanotherapeutics for hepatocellular carcinoma. Journal of Controlled Release.

[ref-70] Zailaie SA, Sergi CM (2022). MiR-126 in hepatocellular carcinoma and cholangiocellular carcinoma: a reappraisal with an *in situ* detection of miR-126. Annals of Clinical & Laboratory Science.

[ref-71] Zhang Z, Lin F, Wu W, Jiang J, Zhang C, Qin D, Xu Z (2024). Exosomal microRNAs in lung cancer: a narrative review. Translational Cancer Research.

[ref-72] Zhang Y, Pan Q, Shao Z (2023). Extracellular vesicles derived from cancer-associated fibroblasts carry tumor-promotive microRNA-1228-3p to enhance the resistance of hepatocellular carcinoma cells to sorafenib. Human Cell.

[ref-73] Zhang D, Zhang Y, Zhang X, Zhai H, Sun X, Li Y (2021). Circ_0046600 promotes hepatocellular carcinoma progression *via* up-regulating SERBP1 through sequestering miR-1258. Pathology—Research and Practice.

[ref-74] Zhou Z, Li T, Li J, Lin W, Zheng Q (2024). Exosomal transfer of HCC-derived miR-17-5p downregulates NK cell function by targeting RUNX1-NKG2D axis. International Immunopharmacology.

[ref-75] Zhou H, Song T (2021). Conversion therapy and maintenance therapy for primary hepatocellular carcinoma. BioScience Trends.

[ref-76] Zhu C, Jiang L, Xu J, Ren A, Ju F, Shu Y (2020). The urokinase-type plasminogen activator and inhibitors in resectable lung adenocarcinoma. Pathology—Research and Practice.

