# Supplementary figures and images for: CAF-derived miR-642a-3p supports migration, invasion, and EMT of hepatocellular carcinoma cells by targeting SERPINE1
Source: PeerJ. 2024 Nov 11;12:e18428. doi: 10.7717/peerj.18428 (PMC11562775; doi:10.7717/peerj.18428)

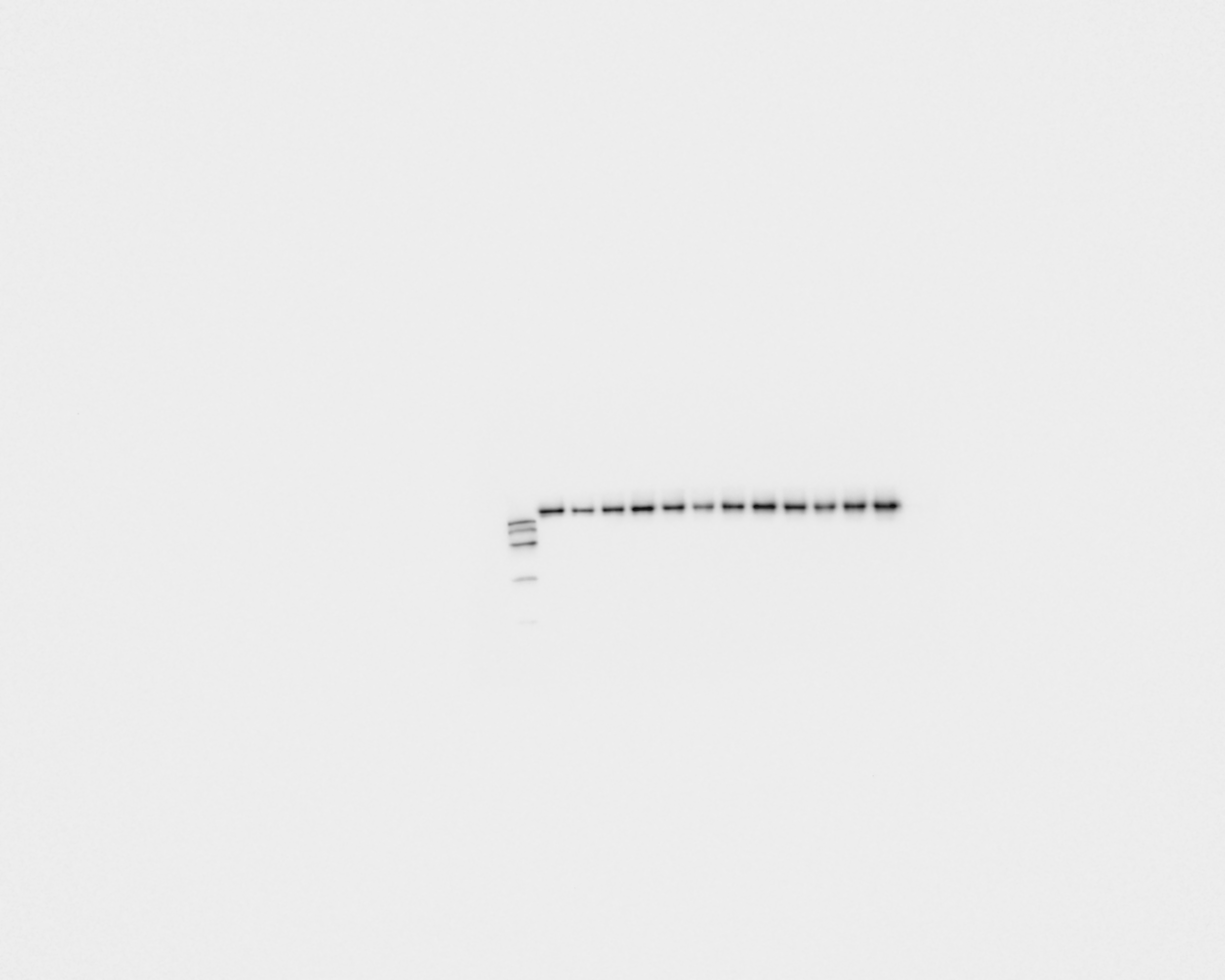

Supplement: Supplemental Information 8 [file peerj-12-18428-s008.zip › E-cadherin.tif]

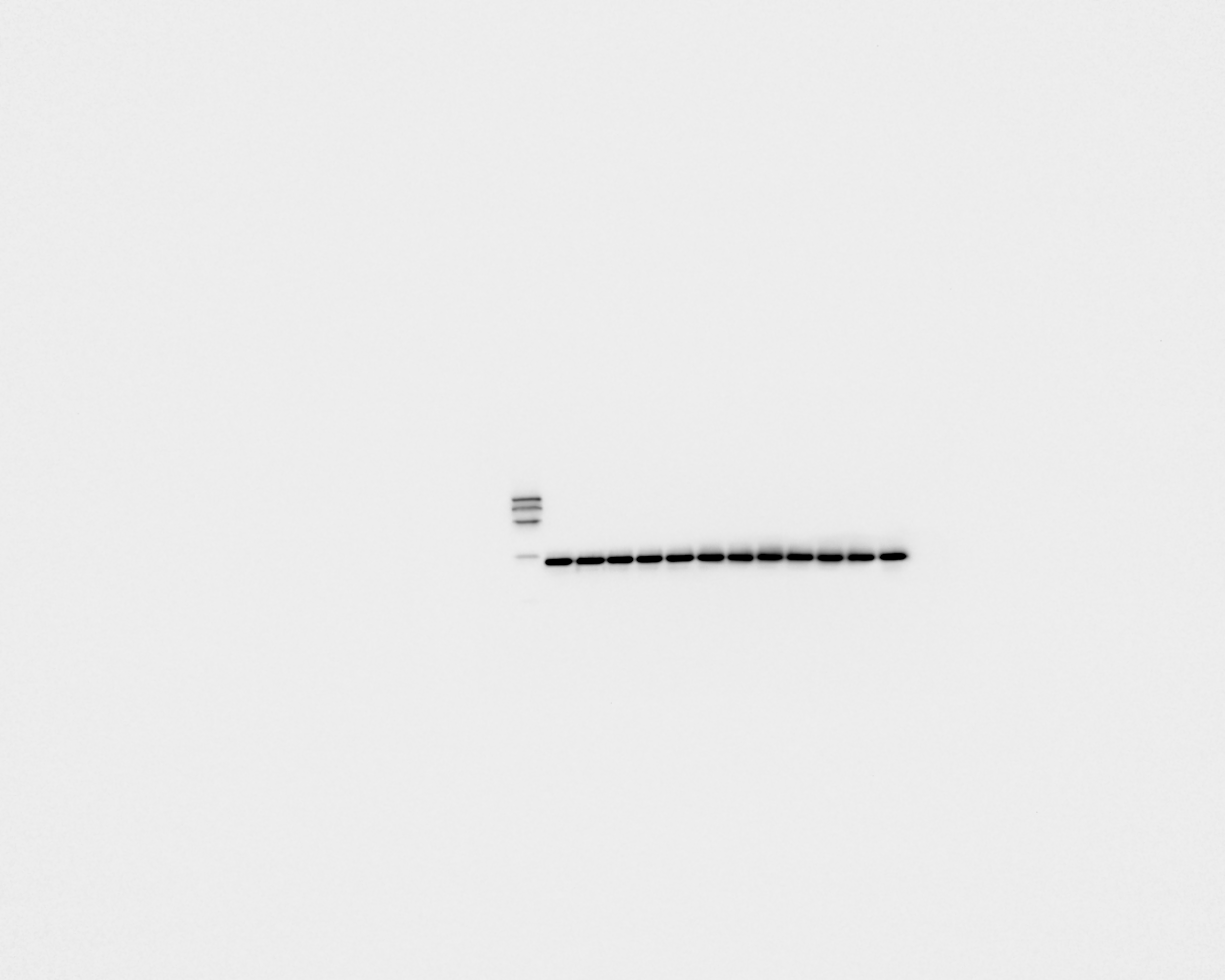

Supplement: Supplemental Information 8 [file peerj-12-18428-s008.zip › GAPDH.tif]

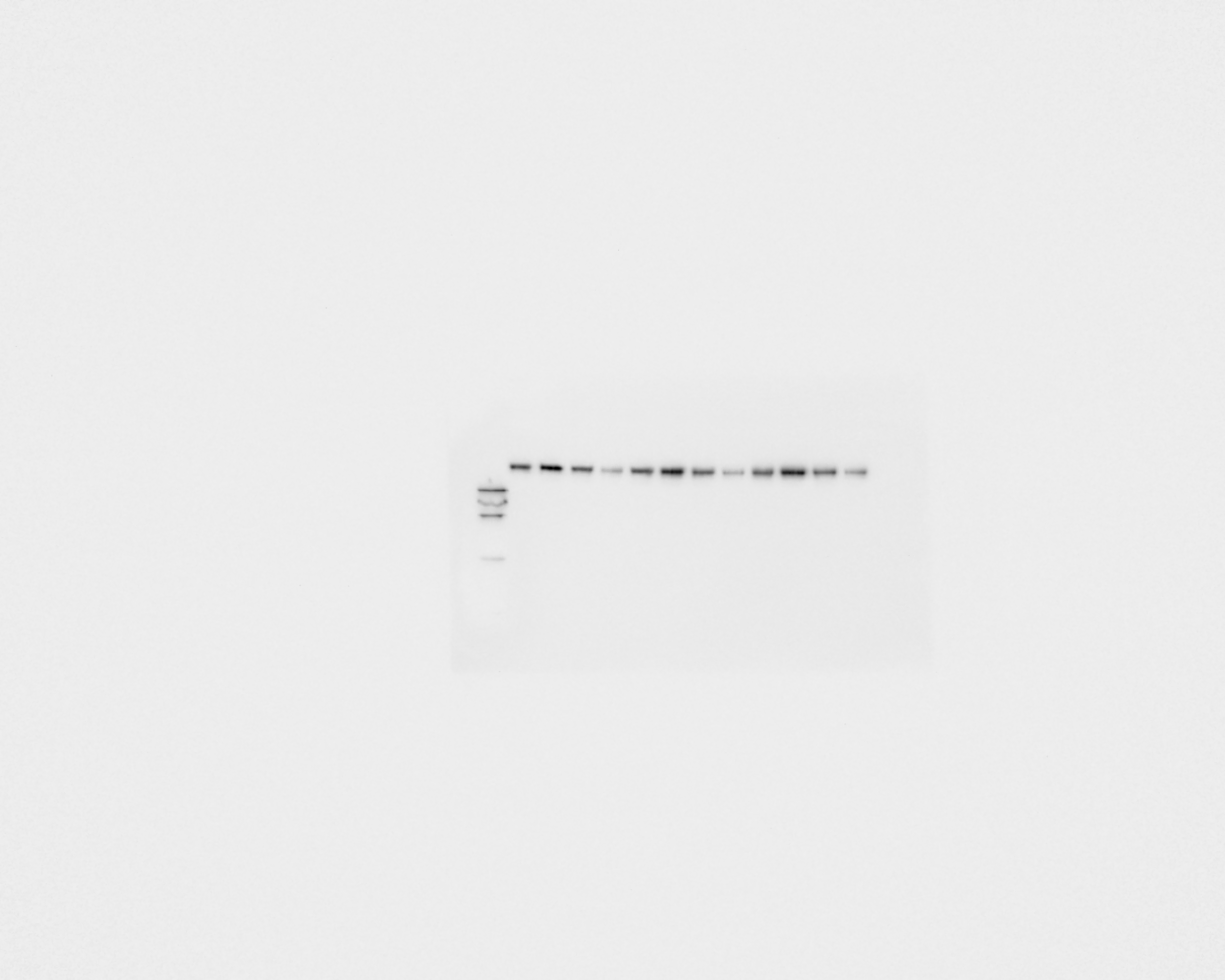

Supplement: Supplemental Information 8 [file peerj-12-18428-s008.zip › N-cadherin.tif]

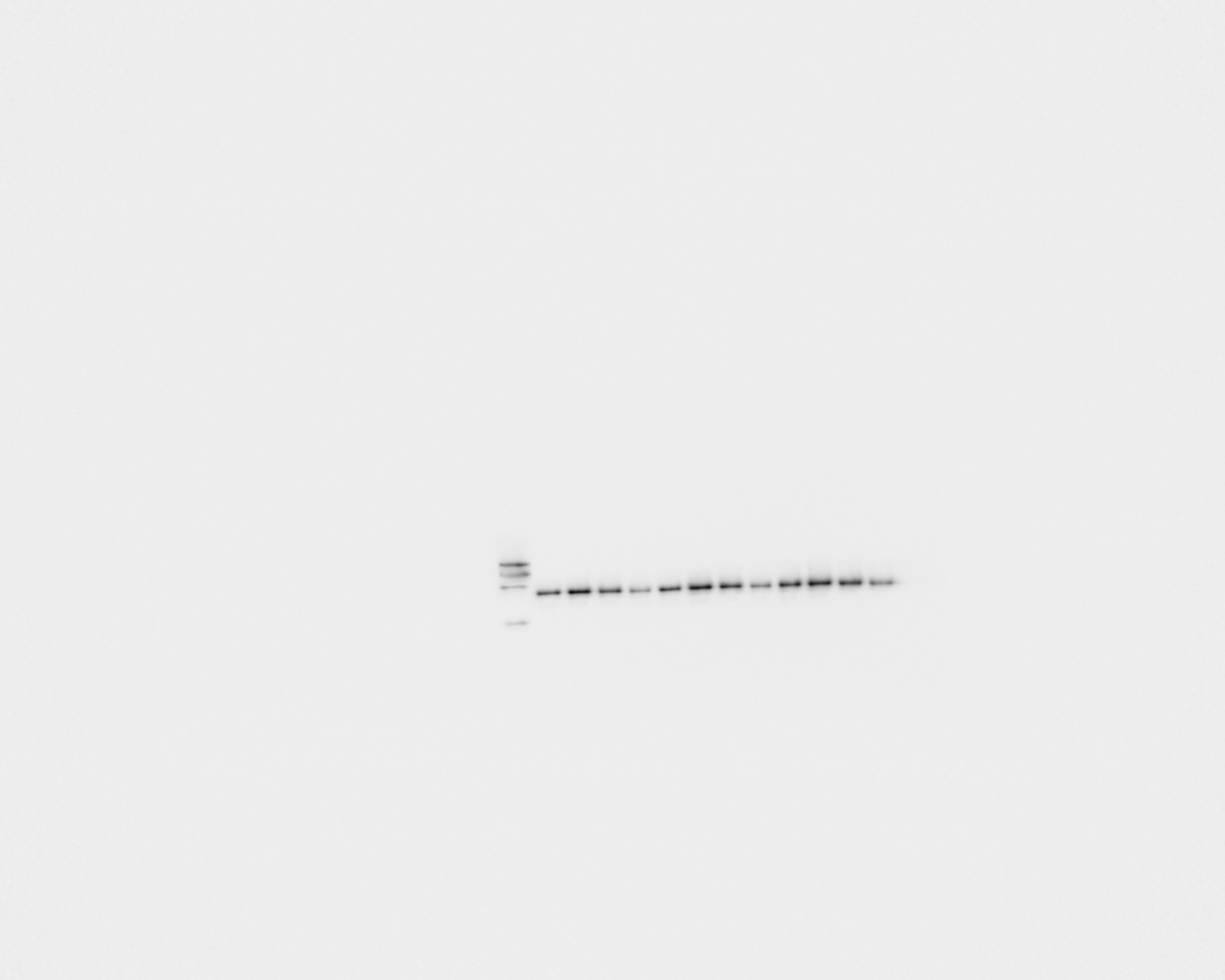

Supplement: Supplemental Information 8 [file peerj-12-18428-s008.zip › Vimentin.tif]

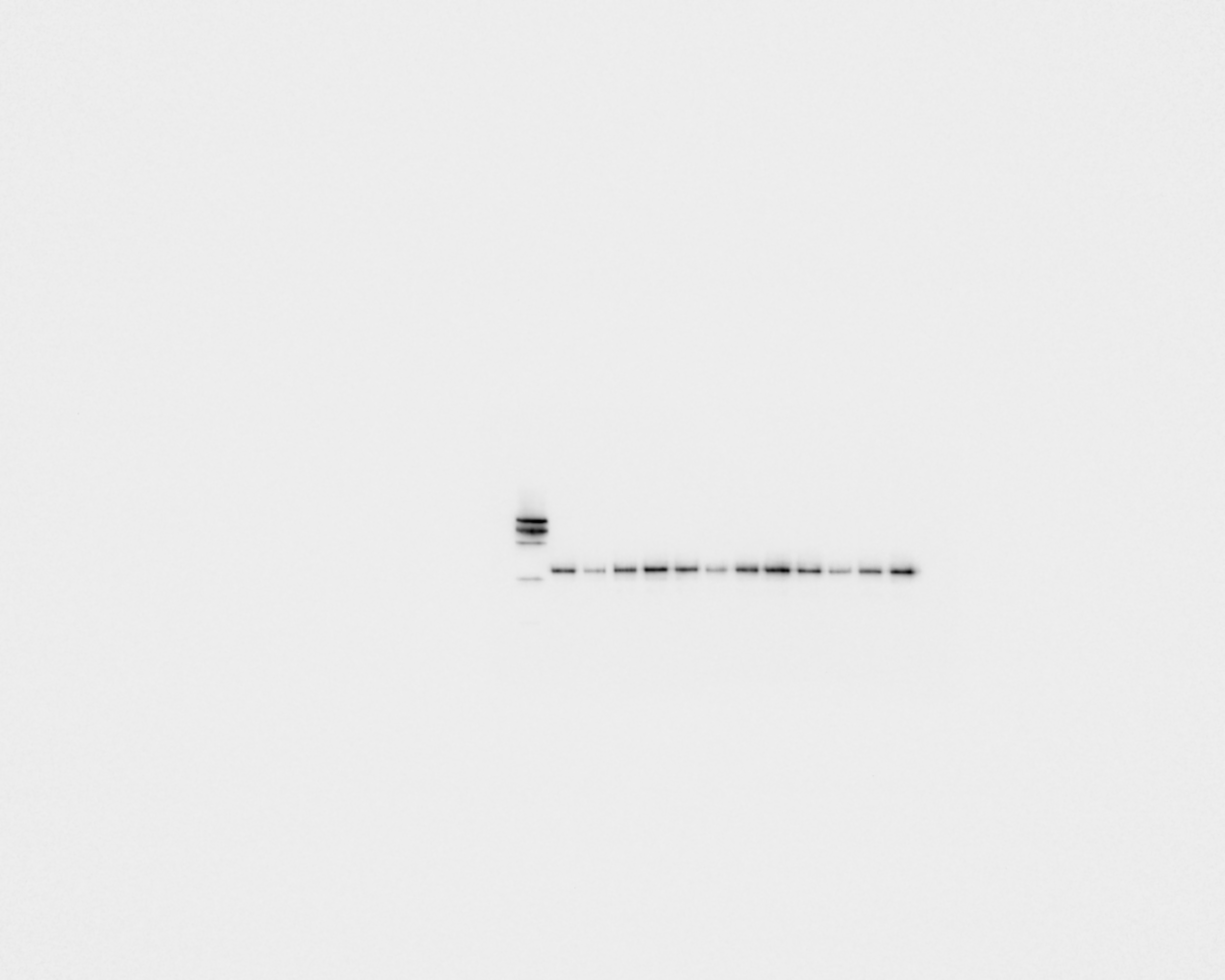

Supplement: Supplemental Information 8 [file peerj-12-18428-s008.zip › serpine1.tif]

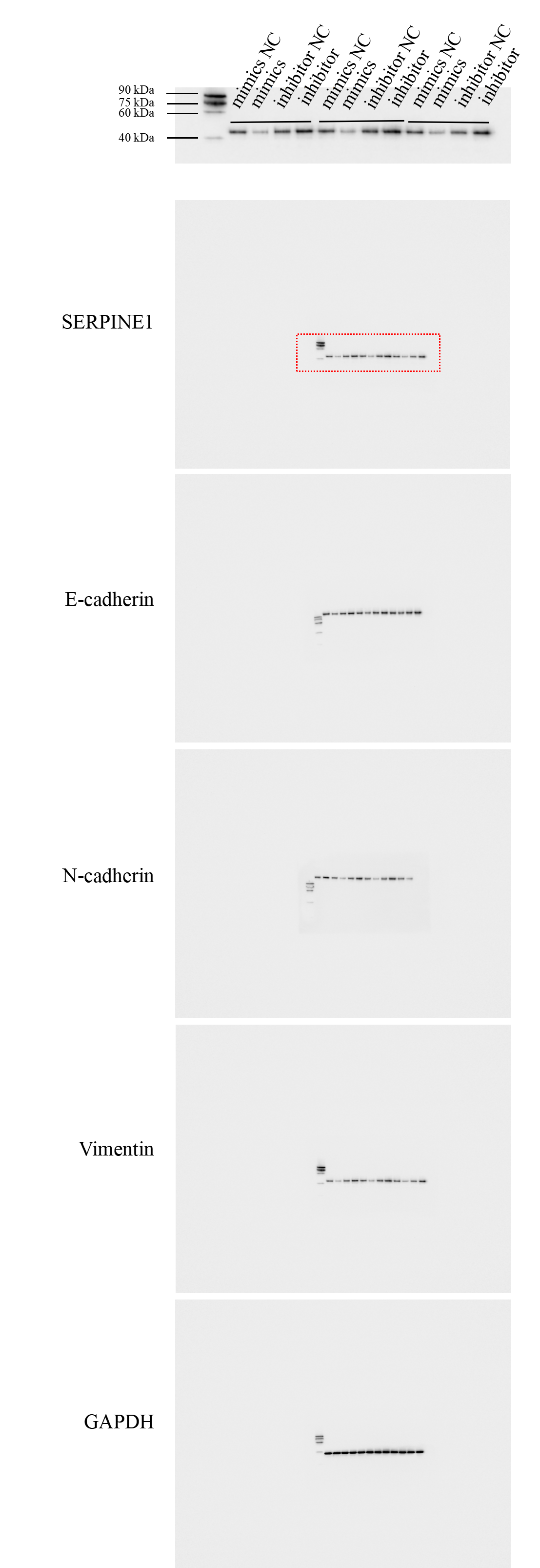

Supplement: Supplemental Information 8 [file peerj-12-18428-s008.zip › Description.png]

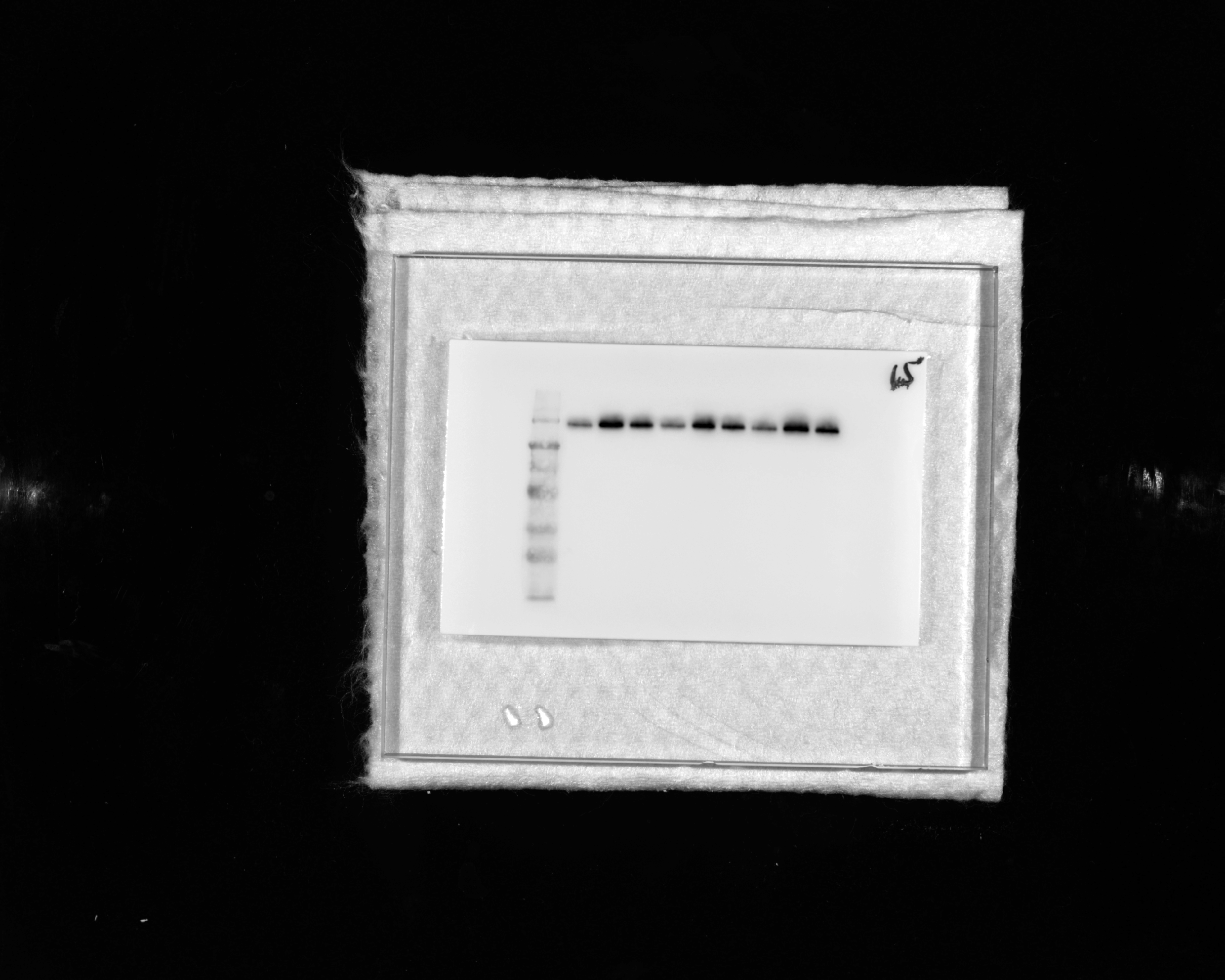

Supplement: Supplemental Information 9 [file peerj-12-18428-s009.zip › E-cadherin.tif]

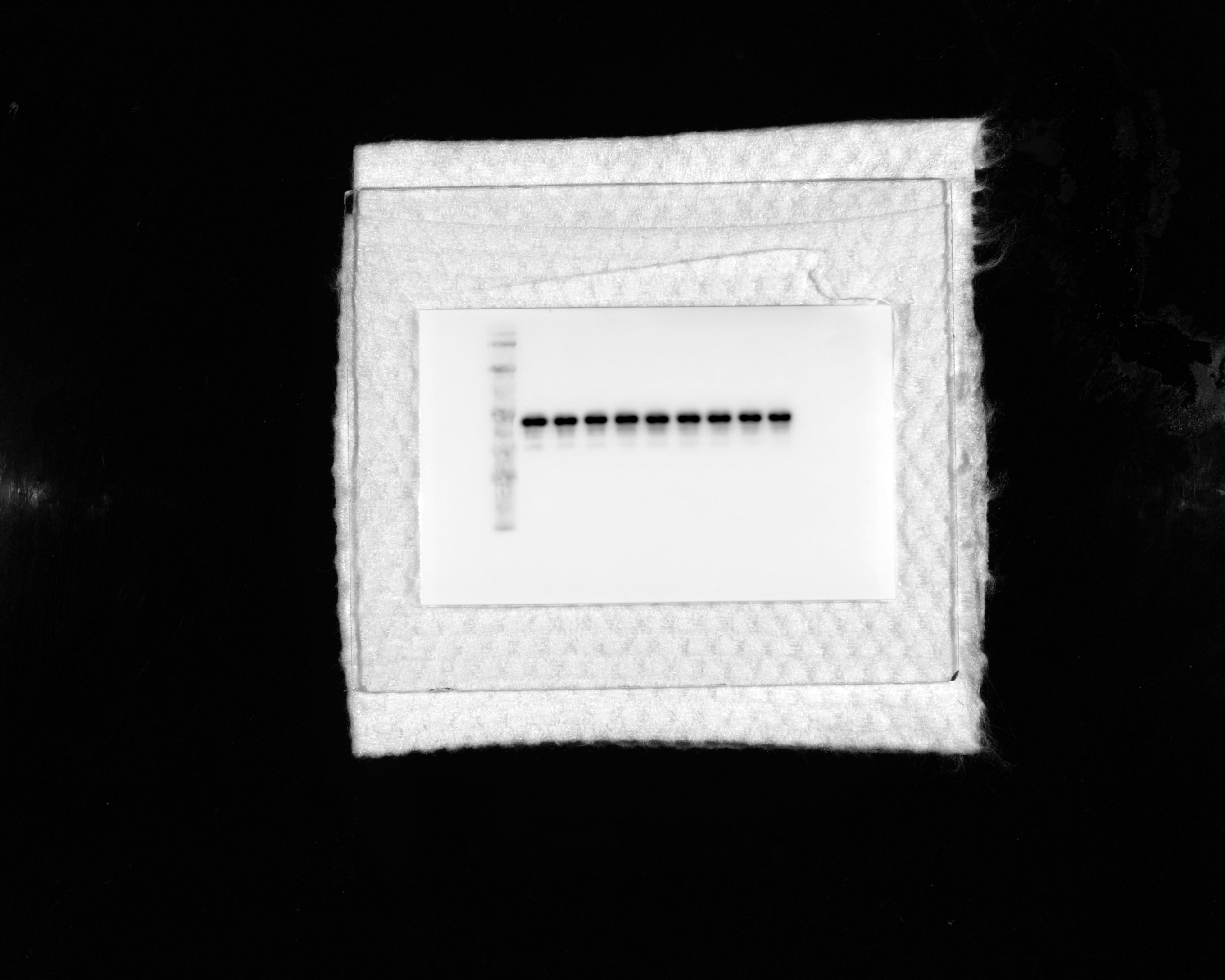

Supplement: Supplemental Information 9 [file peerj-12-18428-s009.zip › GAPDH.tif]

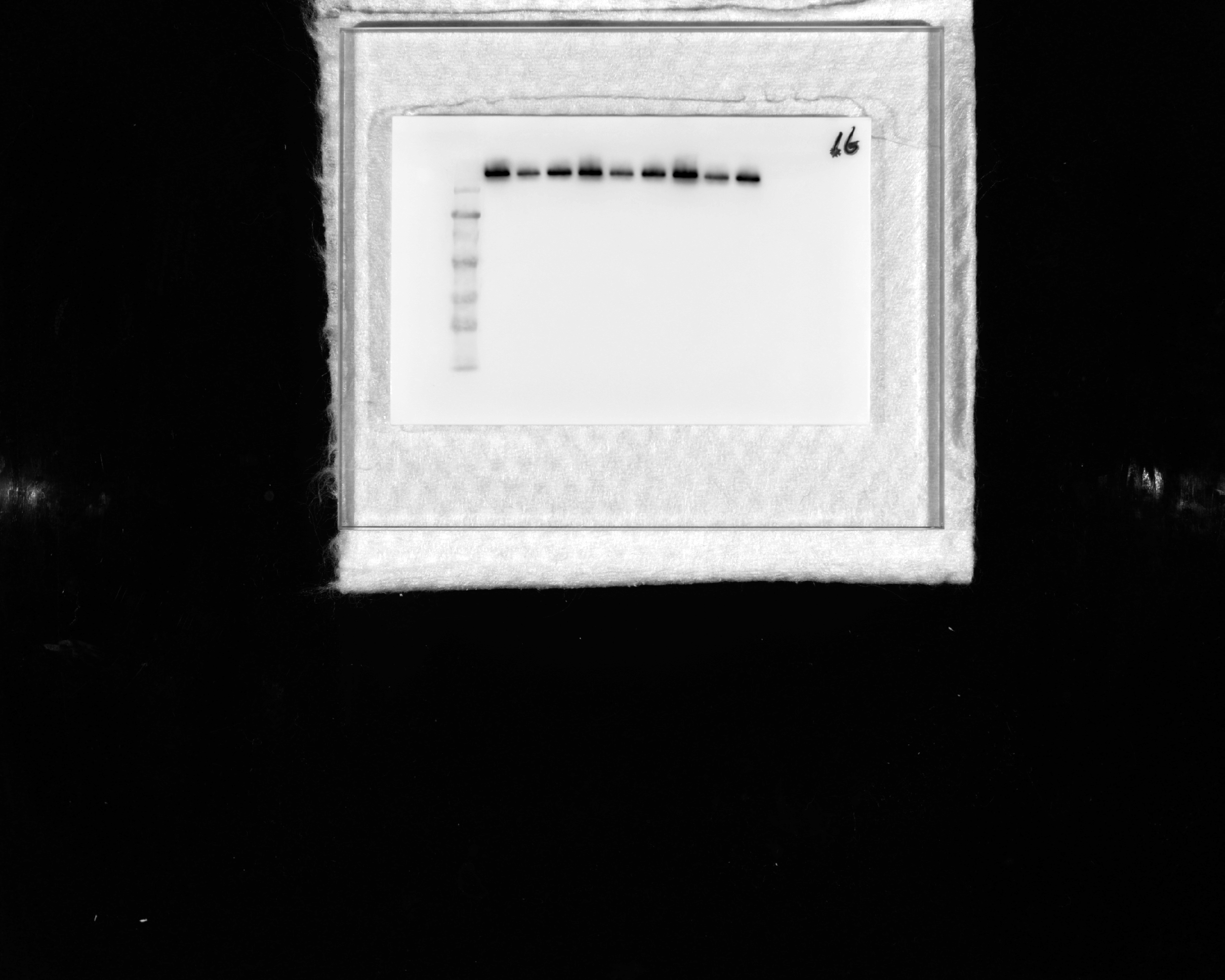

Supplement: Supplemental Information 9 [file peerj-12-18428-s009.zip › N-cadherin.tif]

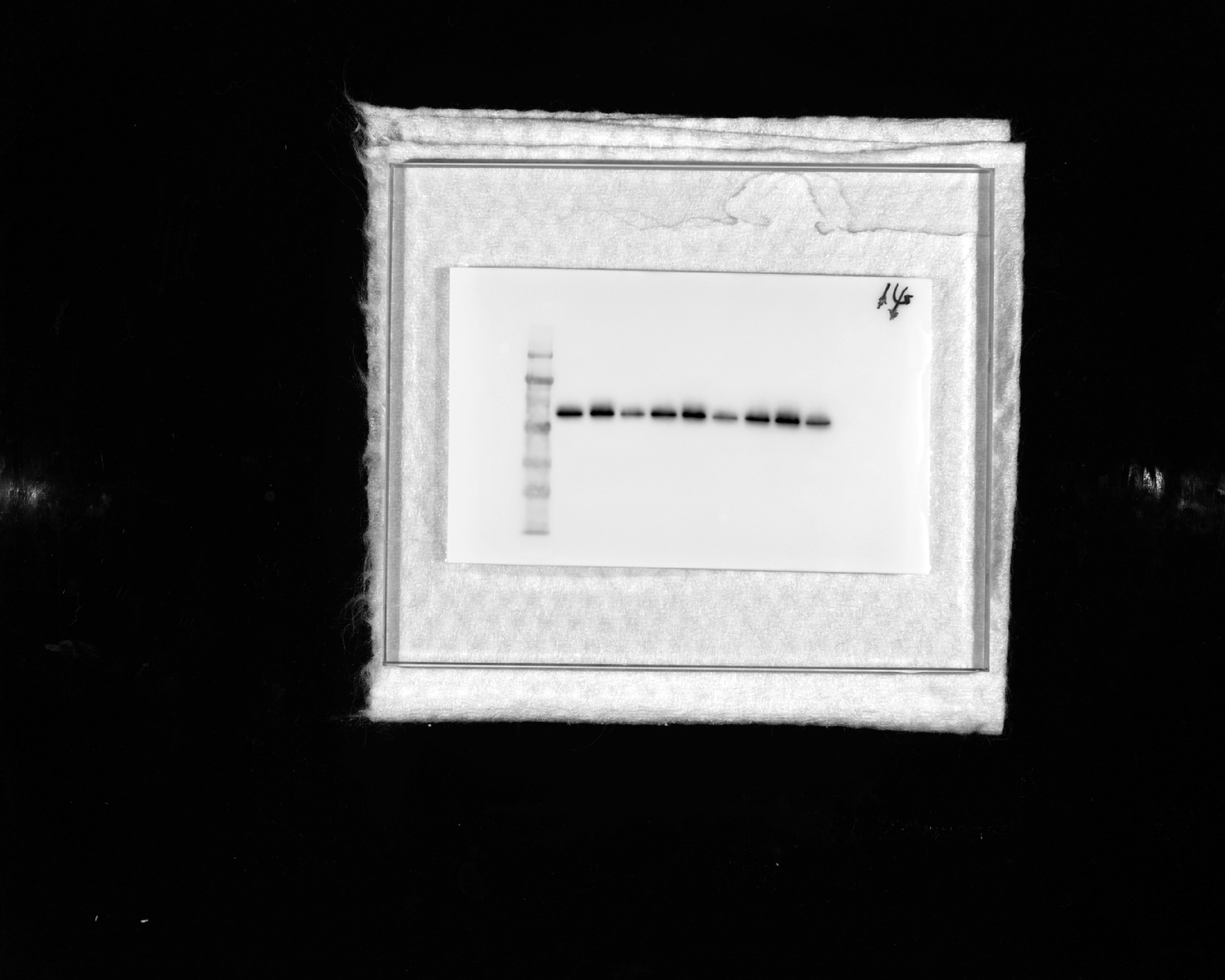

Supplement: Supplemental Information 9 [file peerj-12-18428-s009.zip › serpine1.tif]

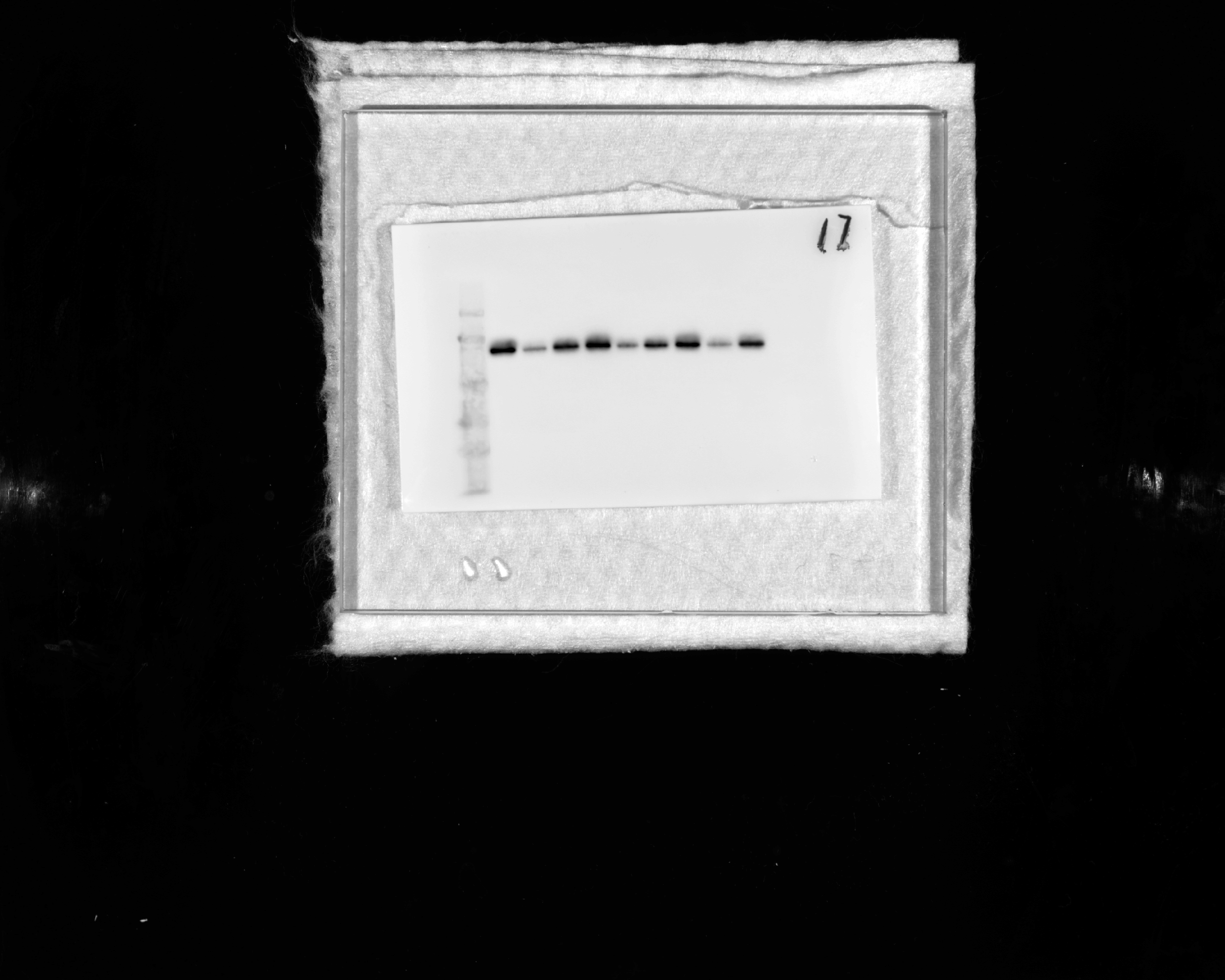

Supplement: Supplemental Information 9 [file peerj-12-18428-s009.zip › vimentin.tif]

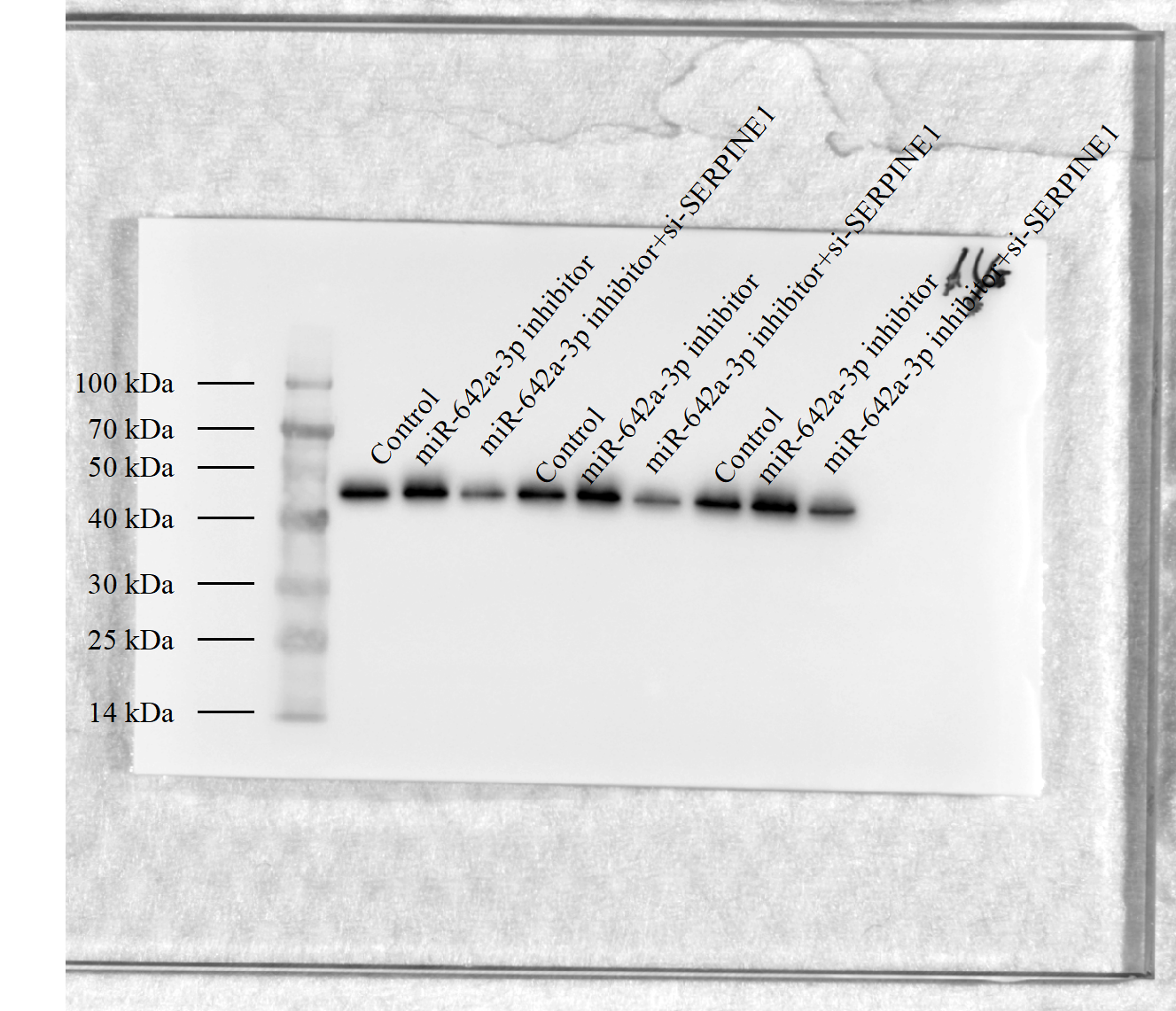

Supplement: Supplemental Information 9 [file peerj-12-18428-s009.zip › Description.png]

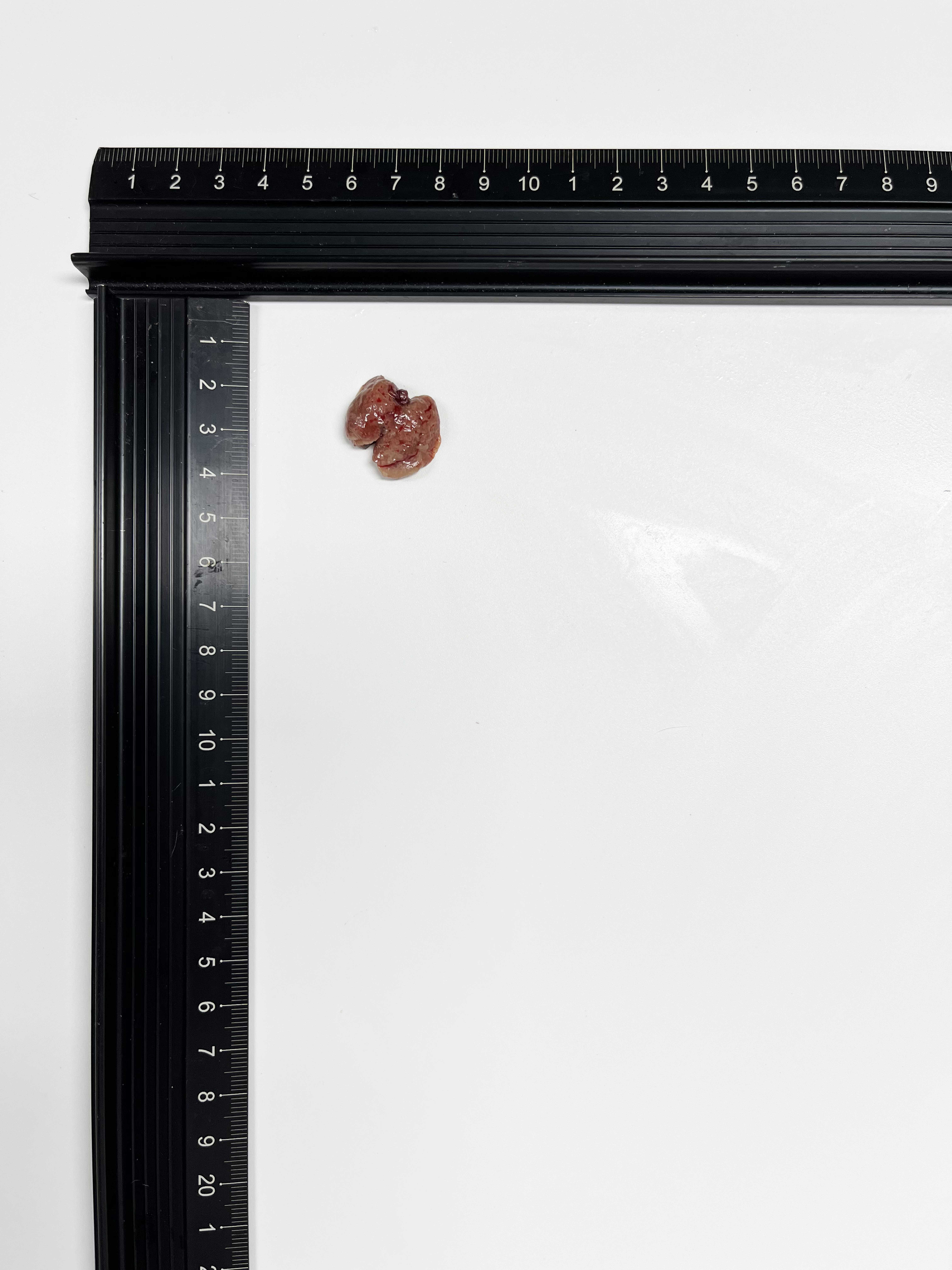

Supplement: Supplemental Information 10 [file peerj-12-18428-s010.zip › Figure 4a/AAV-NC.jpg]

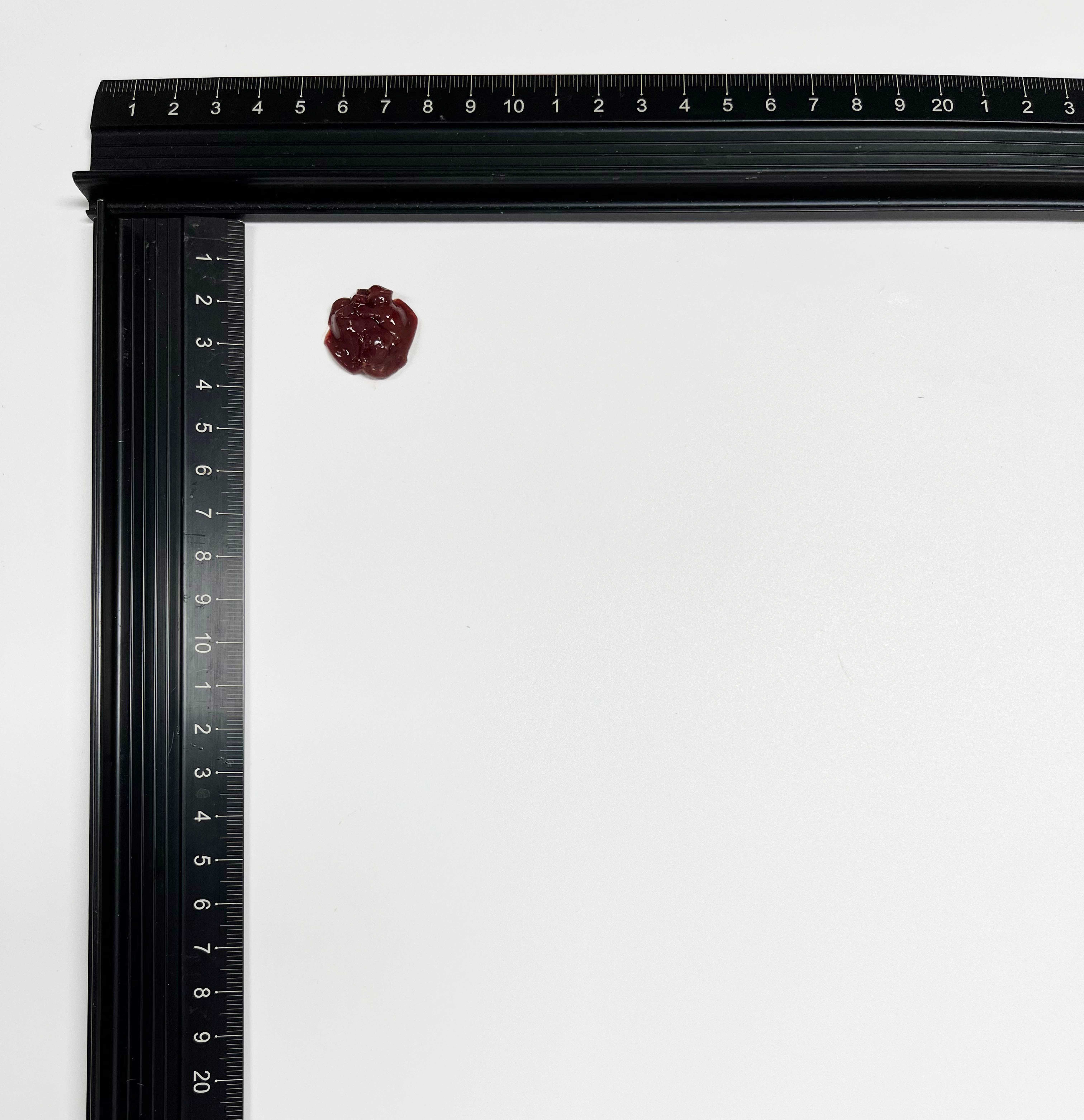

Supplement: Supplemental Information 10 [file peerj-12-18428-s010.zip › Figure 4a/AAV-miRNA.jpg]

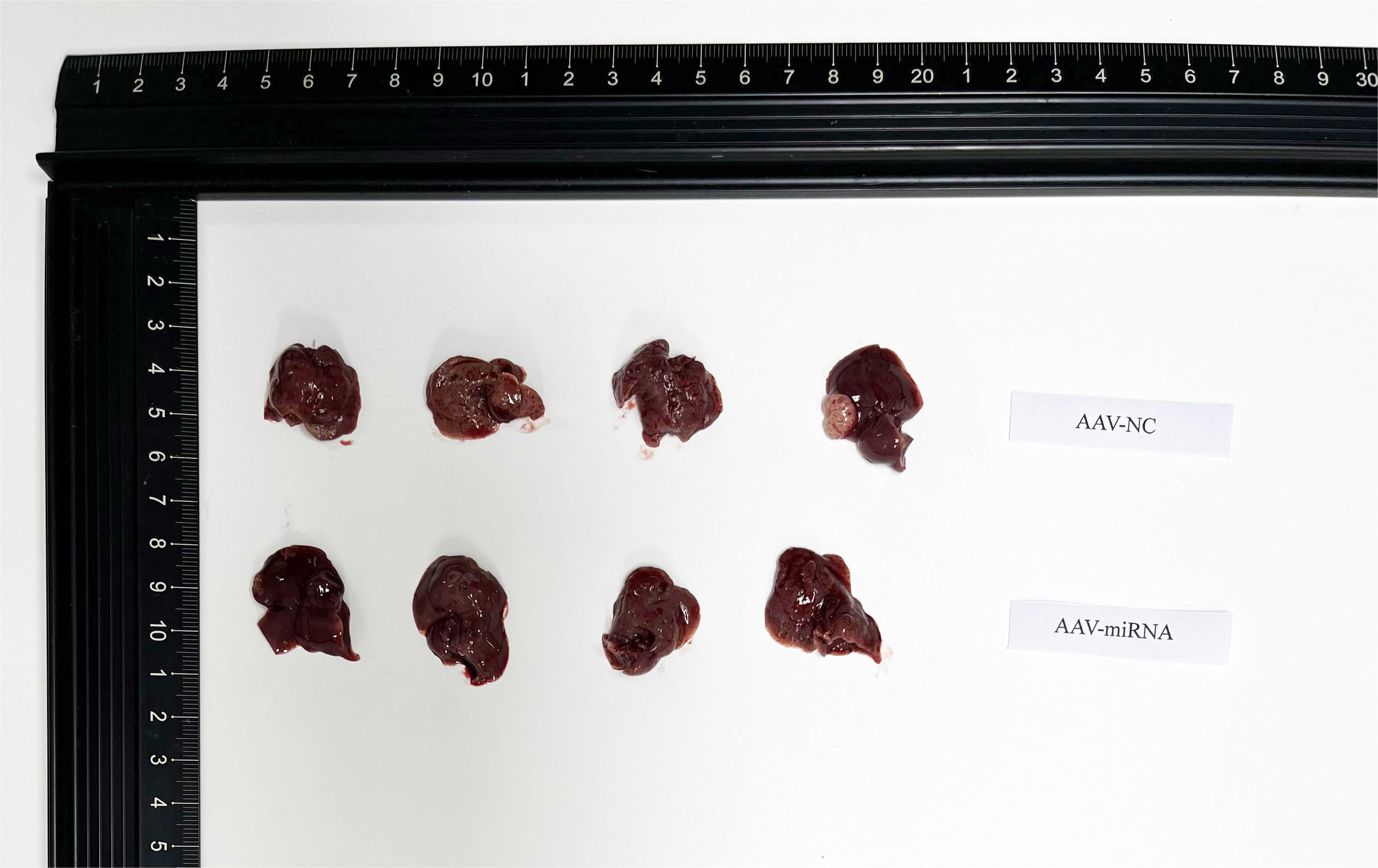

Supplement: Supplemental Information 10 [file peerj-12-18428-s010.zip › Figure 4a/liver.jpg]

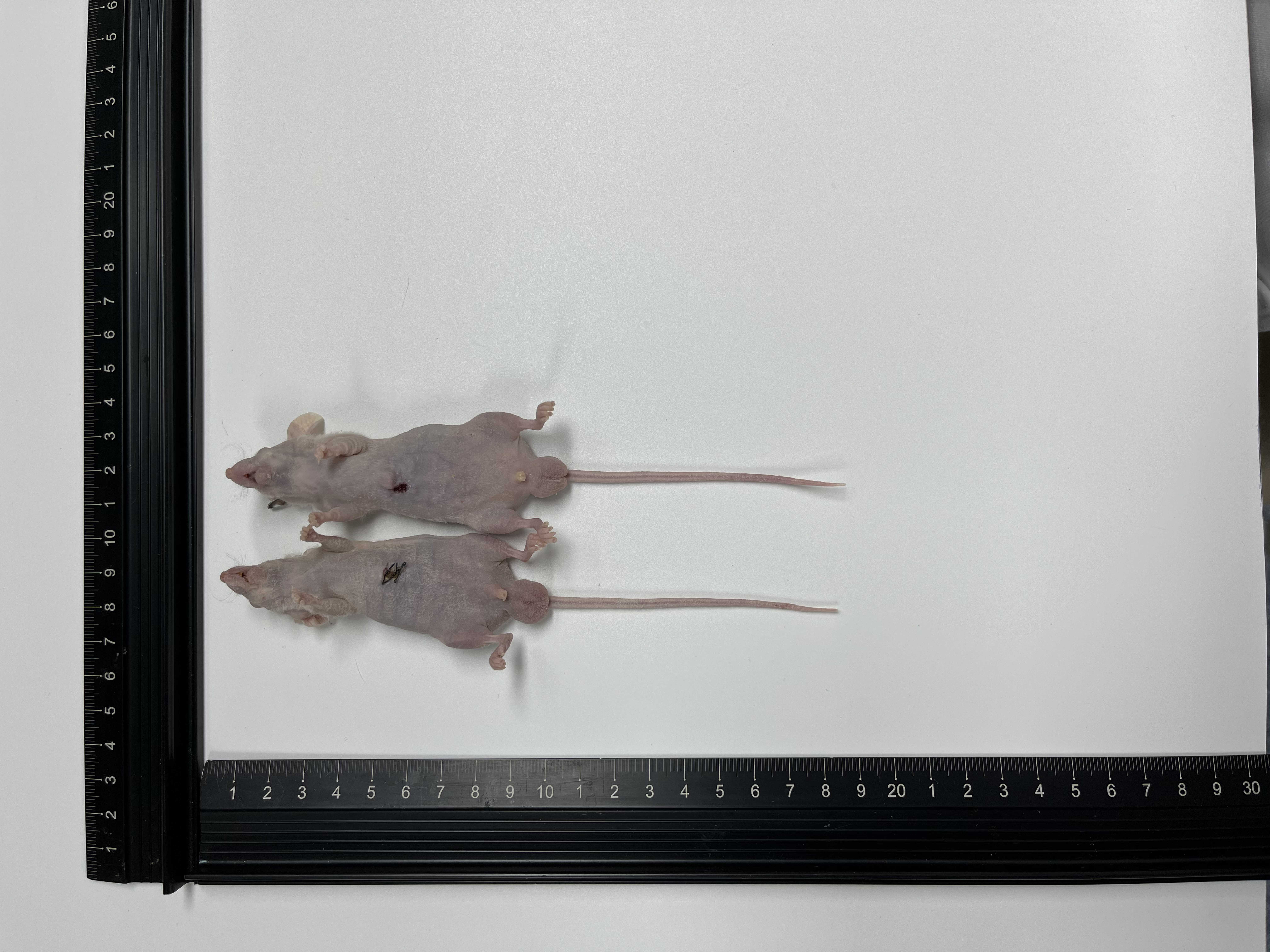

Supplement: Supplemental Information 10 [file peerj-12-18428-s010.zip › Figure 4a/model-1.jpg]

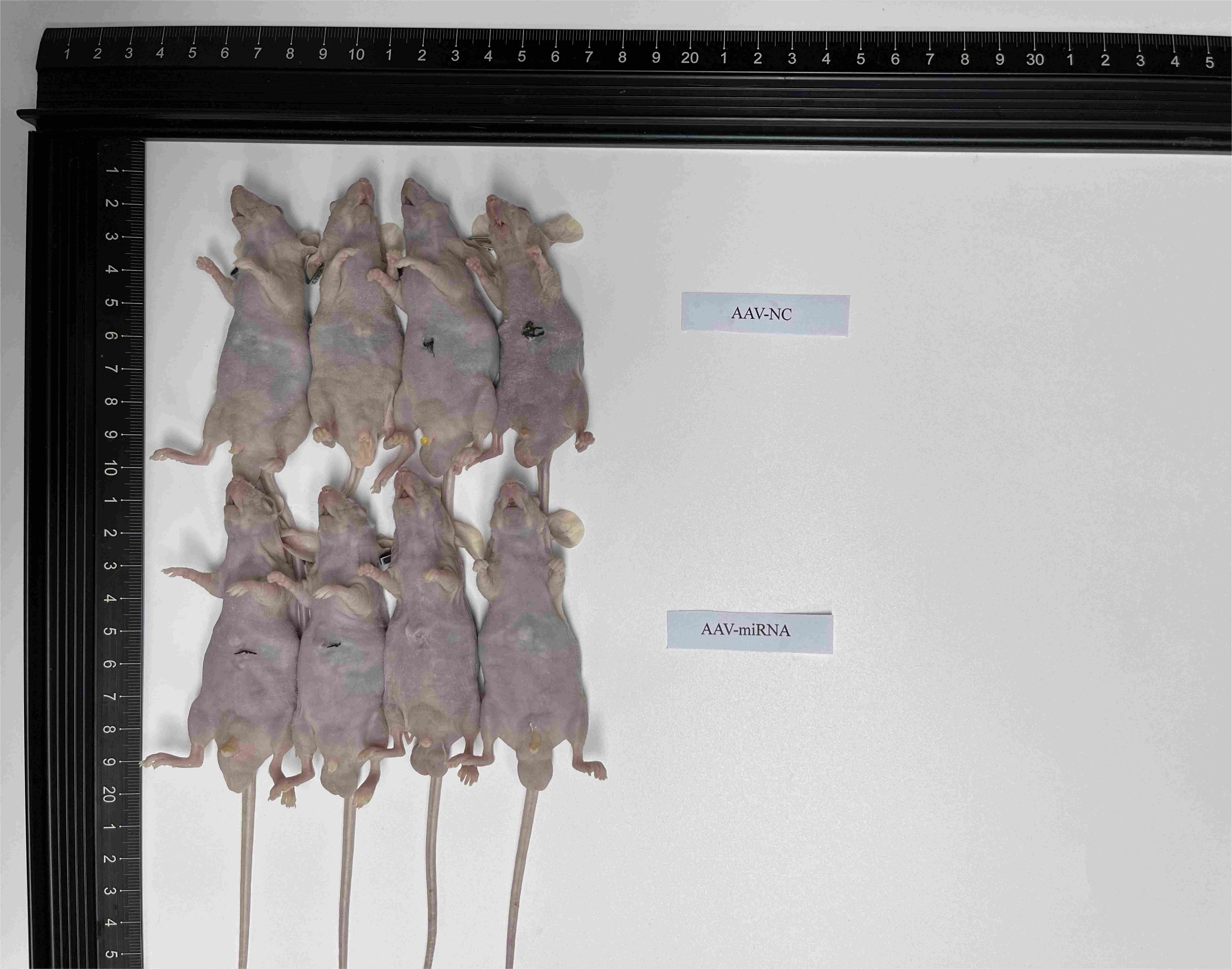

Supplement: Supplemental Information 10 [file peerj-12-18428-s010.zip › Figure 4a/model-2.jpg]

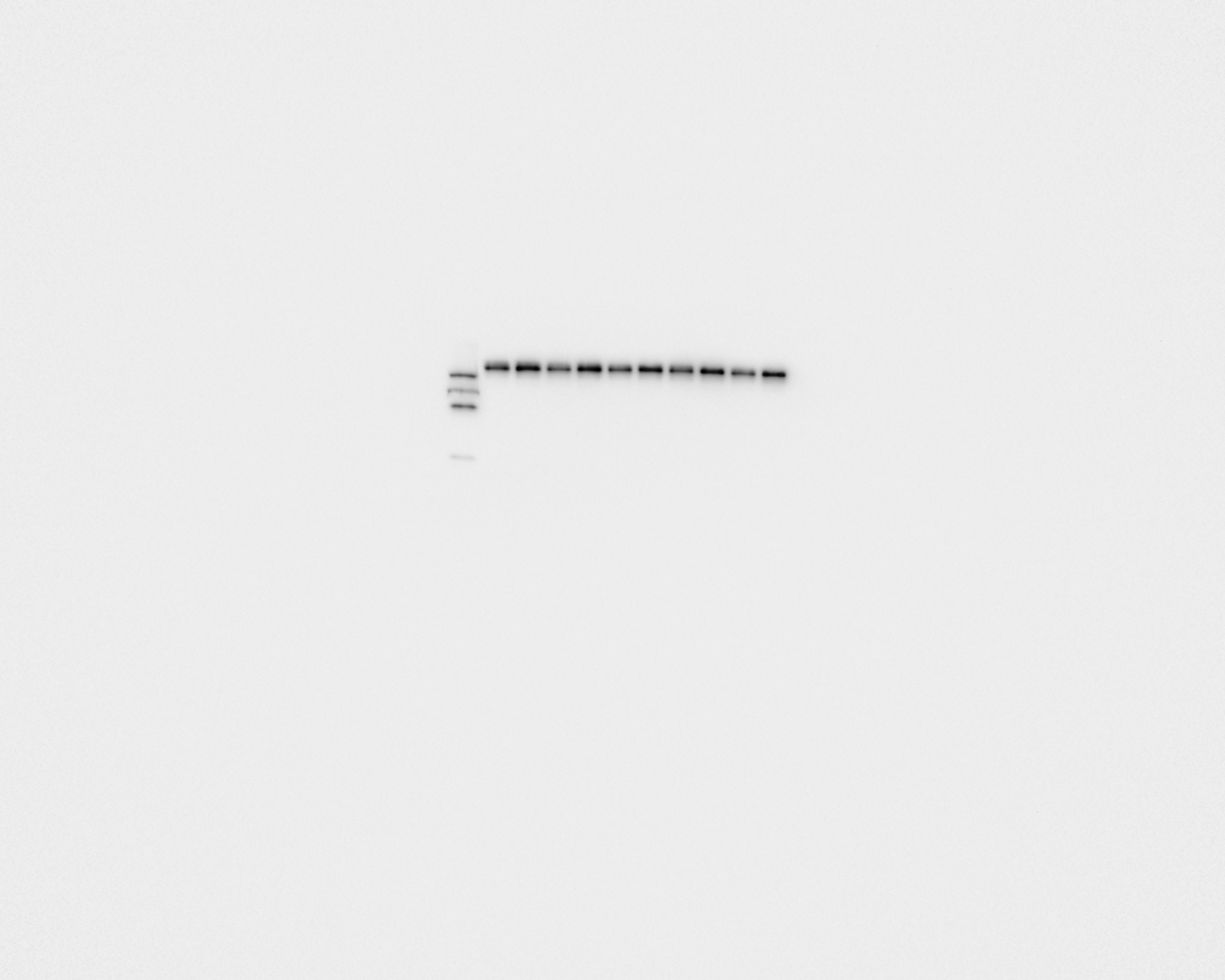

Supplement: Supplemental Information 12 [file peerj-12-18428-s012.zip › E-CAD.tif]

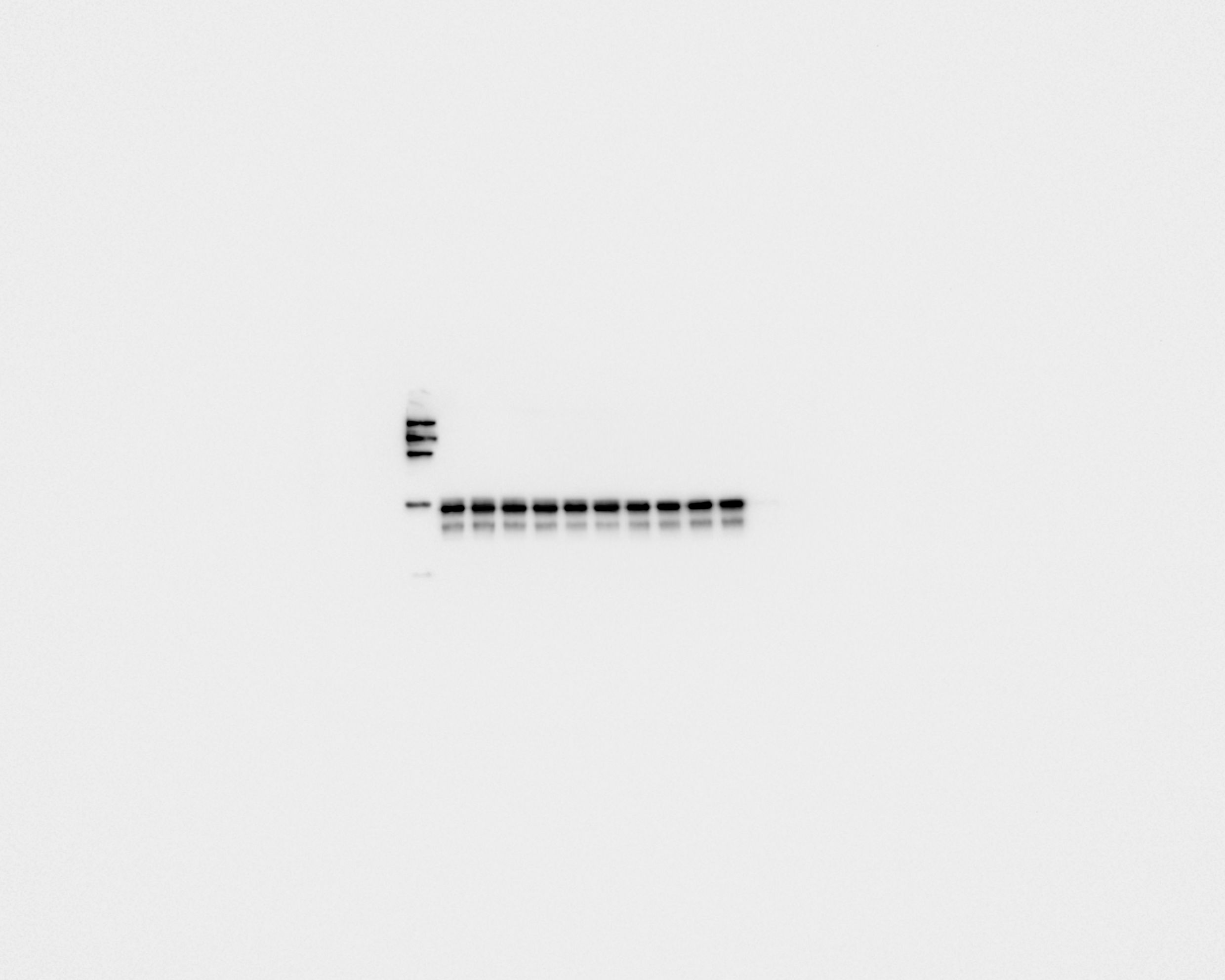

Supplement: Supplemental Information 12 [file peerj-12-18428-s012.zip › GAPDH.tif]

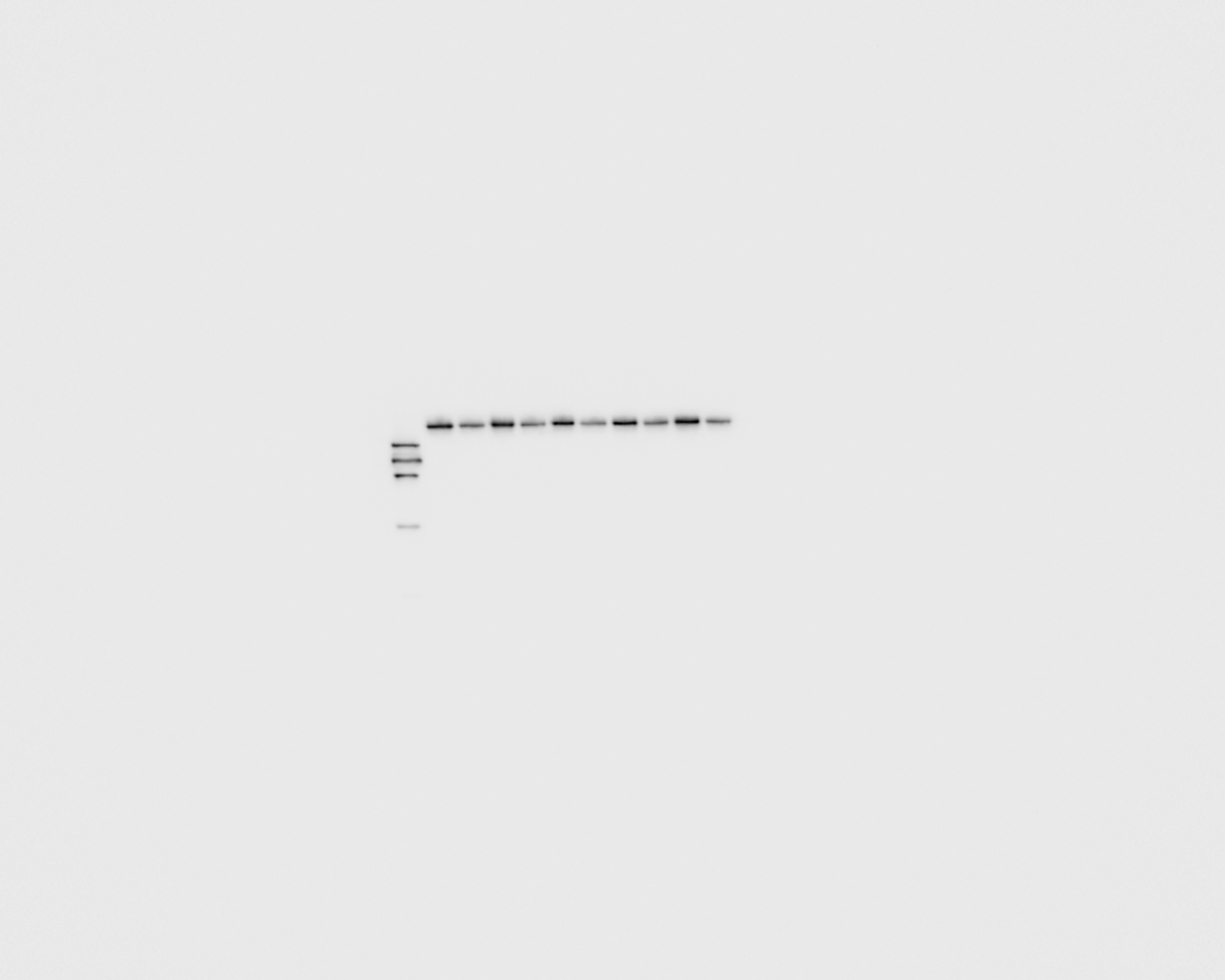

Supplement: Supplemental Information 12 [file peerj-12-18428-s012.zip › N-CAD.tif]

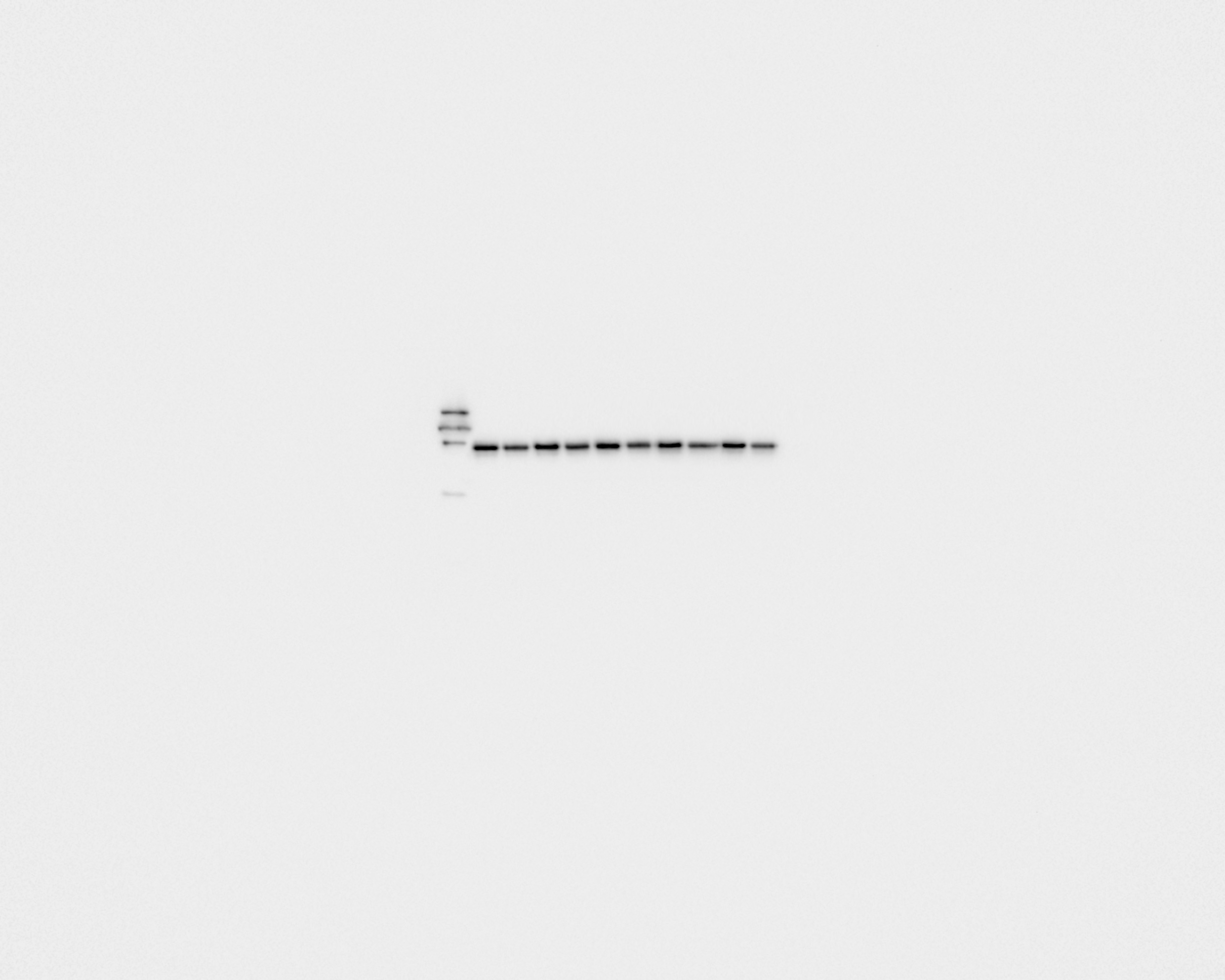

Supplement: Supplemental Information 12 [file peerj-12-18428-s012.zip › VIM.tif]

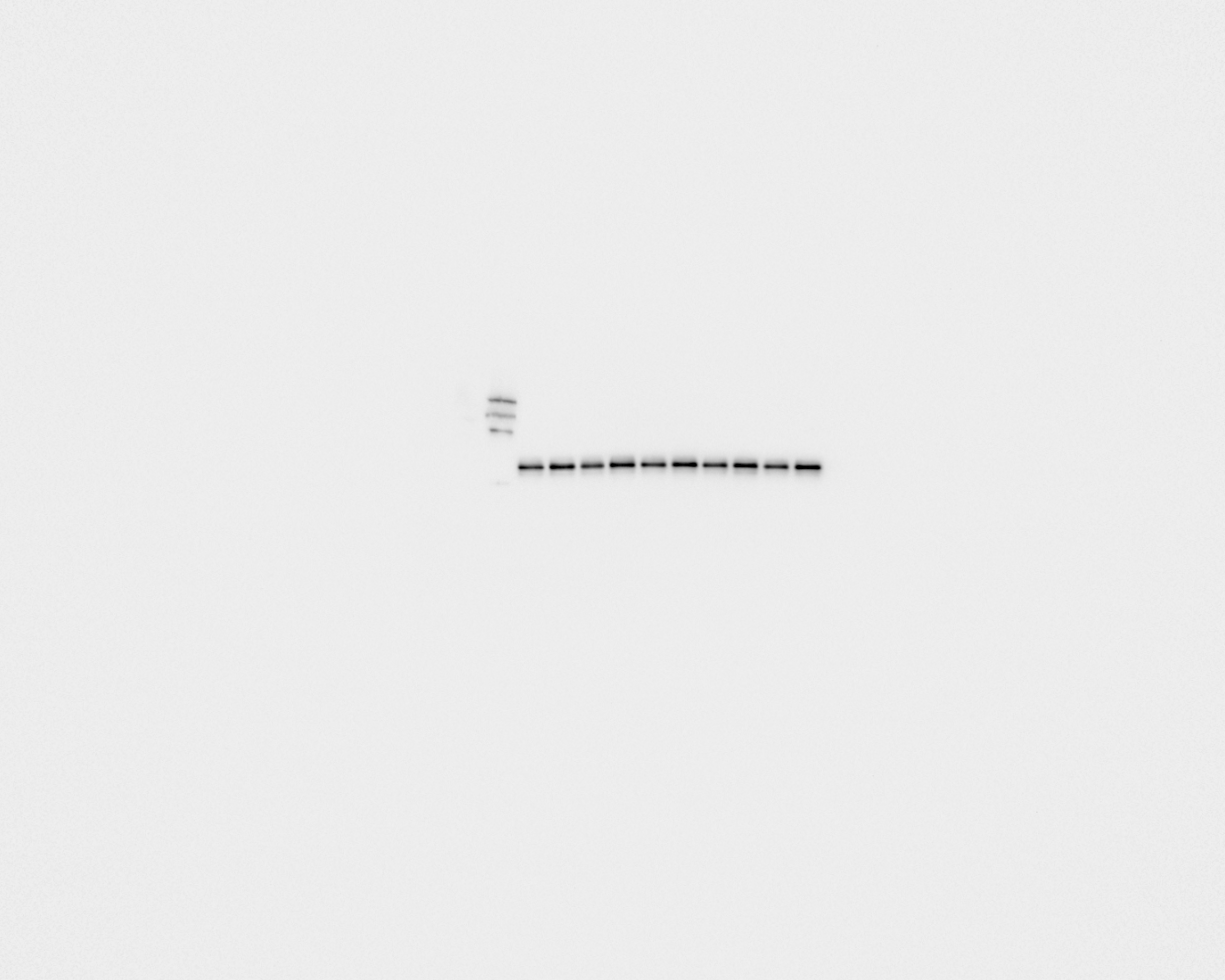

Supplement: Supplemental Information 12 [file peerj-12-18428-s012.zip › serpne1.tif]

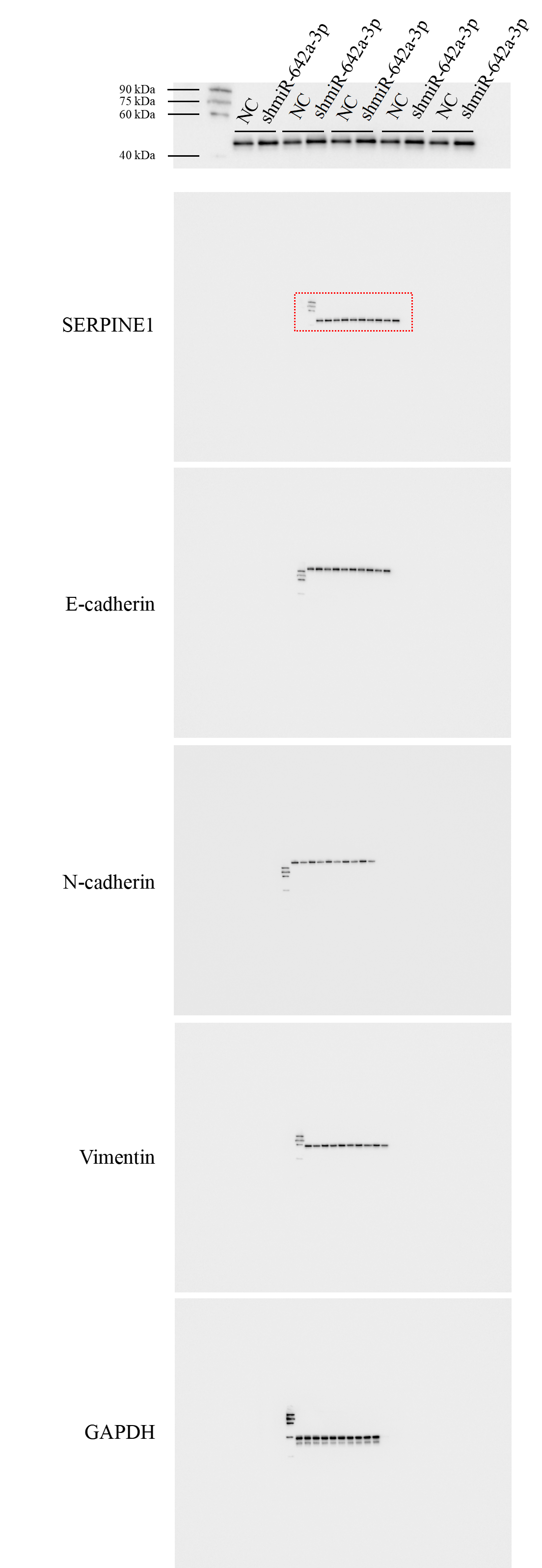

Supplement: Supplemental Information 12 [file peerj-12-18428-s012.zip › Description.png]
