# Supplementary material for: CAF-derived miR-642a-3p supports migration, invasion, and EMT of hepatocellular carcinoma cells by targeting SERPINE1
Source: PeerJ. 2024 Nov 11;12:e18428. doi: 10.7717/peerj.18428 (PMC11562775; doi:10.7717/peerj.18428)
Supplement: Supplemental Information 13 — One animal were shown first in each group (n = 5). [file peerj-12-18428-s013.docx]

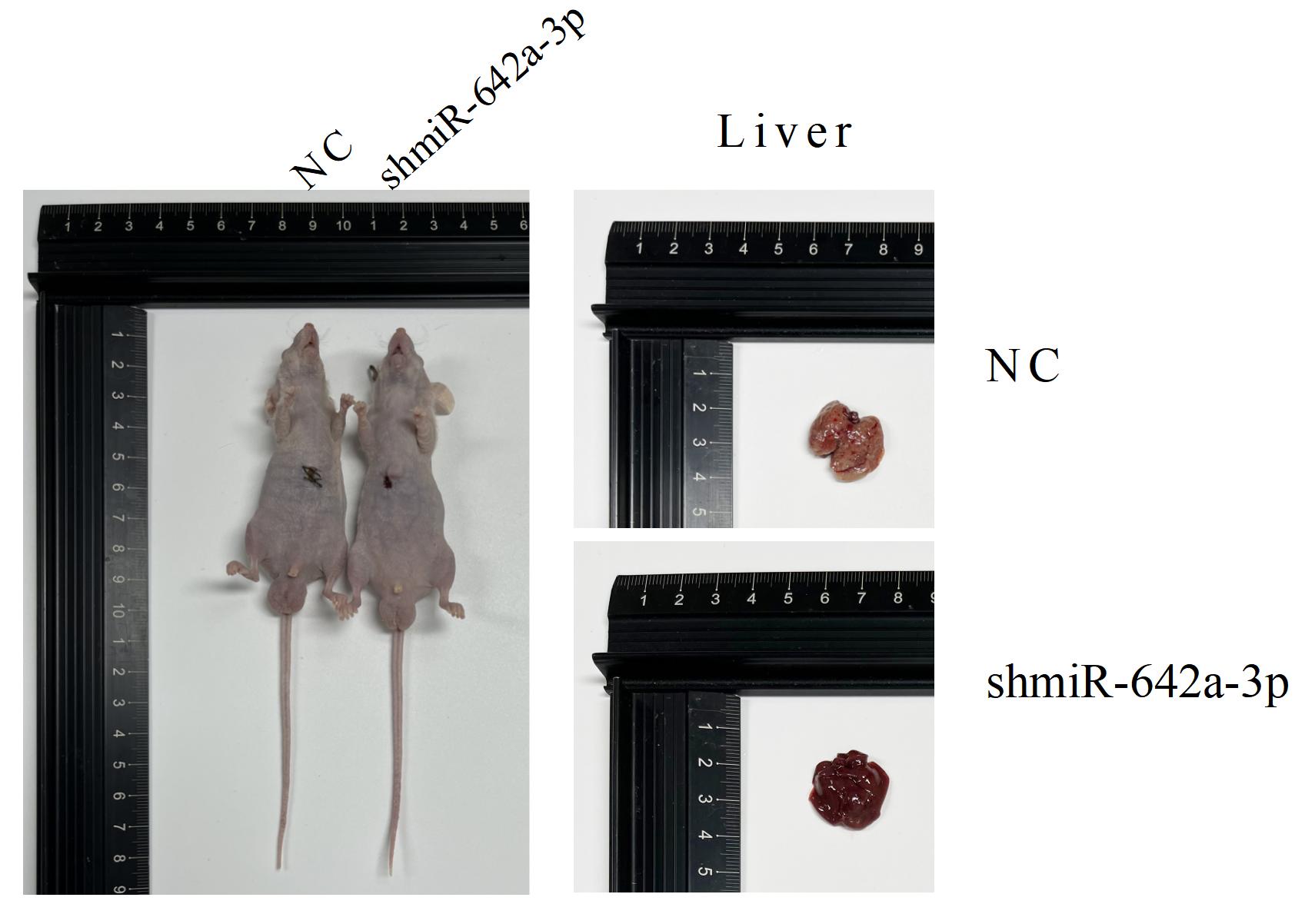


**Supplementary Figure 1** Effect of miR-642a-3p knockdown on growth of orthotopic liver tumors. One animals were shown first in each group (n = 5).
